# Predisposing deleterious variants in the cancer-associated human kinases in the global populations

**DOI:** 10.1371/journal.pone.0298747

**Published:** 2024-04-18

**Authors:** Salman Ahmed Khan, Misbah Anwar, Atia Gohar, Moom R. Roosan, Daniel C. Hoessli, Ambrina Khatoon, Muhammad Shakeel

**Affiliations:** 1 Department of Molecular Medicine (DMM), Dow College of Biotechnology (DCoB), Dow University of Health Sciences (DUHS), Karachi, Pakistan; 2 DOW-DOGANA Advanced Molecular Genetics and Genomics Disease Research and Treatment Center (AMGGDRTC), Dow University of Health Sciences (DUHS), Karachi, Pakistan; 3 Jamil-ur-Rahman Center for Genome Research, Dr. Panjwani Center for Molecular Medicine and Drug Research, International Center for Chemical and Biological Sciences (ICCBS), University of Karachi, Karachi, Pakistan; 4 H.E.J. Research Institute of Chemistry, International Center for Chemical and Biological Sciences (ICCBS), University of Karachi, Karachi, Pakistan; 5 Department of Pharmacy Practice, Chapman University School of Pharmacy Harry and Diane Rinker Health Science Campus, Irvine, CA, United States of America; 6 Department of Molecular Medicine, Ziauddin University, Karachi, Pakistan; University of Michigan Medical School, UNITED STATES

## Abstract

Human kinases play essential and diverse roles in the cellular activities of maintaining homeostasis and growth. Genetic mutations in the genes encoding the kinases (or phosphotransferases) have been linked with various types of cancers. In this study, we cataloged mutations in 500 kinases genes in >65,000 individuals of global populations from the Human Genetic Diversity Project (HGDP) and ExAC databases, and assessed their potentially deleterious impact by using the *in silico* tools SIFT, Polyphen2, and CADD. The analysis highlighted 35 deleterious non-synonymous SNVs in the ExAC and 5 SNVs in the HGDP project. Notably, a higher number of deleterious mutations was observed in the Non-Finnish Europeans (26 SNVs), followed by the Africans (14 SNVs), East Asians (13 SNVs), and South Asians (12 SNVs). The gene set enrichment analysis highlighted NTRK1 and FGFR3 being most significantly enriched among the kinases. The gene expression analysis revealed over-expression of NTRK1 in liver cancer, whereas, FGFR3 was found over-expressed in lung, breast, and liver cancers compared to their expression in the respective normal tissues. Also, 13 potential drugs were identified that target the NTRK1 protein, whereas 6 potential drugs for the FGFR3 target were identified. Taken together, the study provides a framework for exploring the predisposing germline mutations in kinases to suggest the underlying pathogenic mechanisms in cancers. The potential drugs are also suggested for personalized cancer management.

## 1. Introduction

Among all the biological molecules, proteins are the key components involved in diverse cellular processes such as metabolism and signal transduction pathways. A specialized category of phosphotransferase proteins called “kinases” are essential for the cellular vital activities through a chemically reversible and swift phenomenon called protein phosphorylation [1–4]. Protein phosphorylation is a post-translational modification managed by specific domains (e.g., the SH2 domain) activating/deactivating many enzymes and receptors by means of kinases and phosphatases, acting as molecular switches [5, 6]. Results from the human genome project (HGP) suggested the presence of at least 518 protein kinases in the human genome [7]. These kinases are involved in phosphorylating at least 70% of the total human proteins [8, 9]. This indicates that each protein kinase and phosphatase involves roughly hundreds of phosphorylation events in complex enzymatic networks.

Several families of kinases, such as the rare but critical tyrosine kinases [10] and the major serine/threonine cycle-dependent kinases [11–13], aurora kinases [14], mTOR [15], and mitogen-activted protein kinases [16], have been reported so far. Despite their normal physiological role, kinase-mediated signaling is well known in various diseases such as cancer, and often causes the disease itself or drives its progression. To detect mutationally activated kinases in cancer signaling pathways, several large-scale cancer genome sequencing projects have been conducted such as the Cancer Genome Atlas (TCGA) [17]. Findings of such projects have played a significant role in the development of inhibitors of oncogenic kinases for cancer therapy since the success of imatinib for Bcr-Abl-positive chronic myeloid leukemia (CML) patients and gastrointestinal stromal tumors (GIST) [18, 19]. Besides oncological issues, kinase dysregulation has also been revealed in other disorders, including metabolic (in particular diabetes), immune, neurological and infectious diseases [20–23].

Given the key importance of kinases in cellular signaling pathways involved in human cancers, identifying and profiling functional missense mutations is a rational approach to designing better diagnostic and therapeutic approaches. With recent advances in the high-throughput sequencing technologies, a large number of genetic variations have been identified. The identification of pathological genetic variants has proven crucial in gene level studies and formulating targeted therapies. However, efficient and effective identification of functionally significant genetic variations associated with the diseased status is a time consuming as well as an expensive task. For this purpose, various bioinformatics tools, designed on the basis of recent findings in protein structure and evolutionary biology may prove useful in predicting the functional importance of genetic mutations [24–27]. Over the past few years, several in silico studies have been attempted to screen the alteration within the protein coding regions of genes, and have highlighted the efficiency of such bioinformatics tools to be efficient and effective platforms for indicating which role these mutations have in the pathology of diseases [28–30].

Non-synonymous single nucleotide polymorphisms (nsSNPs) are the simplest form of genetic variations occurring in the coding region and alter the encoded amino acid in the resultant protein [31]. The nsSNPs-induced alteration in the encoded amino acid may further result in changing the physiochemical properties of native proteins and disrupting their molecular function [32, 33]. Several computational biology tools facilitate the screening of deleterious mutations by taking into account the sequence conservation among species, structural features, and physiochemical properties of proteins [34, 35]. Although there are a great many studies investigating the impact of pathogenic mutations in different cancers cohorts, yet the predisposing germline deleterious mutations for cancers in healthy populations have not been investigated so far. In this study, taking advantage of the publicly available genome-wide mutational database of global populations, such as the Exome Aggregation Consortium (ExAC) [36, 37] and Human Genome Diversity Project (HGDP) [38], we used in an in silico approach to analyze highly deleterious mutated kinases throughout the human kinome relevant to carcinogenesis. The aim of our study is to identify the predisposing pathogenic kinase variants which are likely to be involved in cancer development in global populations.

## 2. Materials and methods

### 2.1 Preparation of gene lists

The genes encoding kinases in humans were obtained from the protein kinase complement of the human genome [7]. The HUGO Gene Nomenclature (HGNC) approved names and symbols of all the genes were confirmed through ENSEMBLE and GENCODE [39, 40]. The genes’ coordinates (start and end positions on the chromosomes) were retrieved from the ENSEMBLE release v94 using the hg38 build.

### 2.2 Database for variants extraction

The complete kinome genes list was passed through two databases of germline mutations i.e., Exome Aggregation Consortium (ExAC, n = 60,706) release 1.0, and Human Genome Diversity Project (HGDP, n = 1,050) [41, 42] using the bcftools v.1.13. The bcftools ‘view’ module was used to subset the variants from the ExAC and HGDP data resulting in a vcf file containing variants in the kinome genes only. The subset variants were re-accessed for their association with cancer through the Catalogue of Somatic Mutation in Cancer (COSMIC) database v87 release [43]. The COSMIC database resulted in two sets of kinase gene variants, specifically, variants in the coding and the non-coding regions.

### 2.3 Variants annotation and filtration

Variants annotated from the COSMIC database were subjected to Combined Annotation Dependent Depletion (CADD) scores for measuring the level of deleteriousness [44]. CADD scores (C-score) threshold were chosen such that variants with C-scores <15 was considered least deleterious, C-scores 15–19.99 were considered deleterious, and variants with C scores ≥20 were considered highly deleterious [45]. For reconfirmation of the deleterious variants, we further annotated these variants with ANNOVAR to determine the SIFT and PolyPhen [46, 47] scores. Finally, the variants predicted as deleterious by the SIFT and PolyPhen2 tools and having CADD C-scores ≥20 were selected for further analysis, and the genes harboring such variants were termed as deleterious genes, as described in our previous study [48].

### 2.4 Gene set enrichment analysis

The underlying biological functions of the 500 kinome genes were evaluated through the PANTHER version 14 [49]. PANTHER is used as a comprehensive resource for the classification of genes according to their evolutionary history and their functions [50, 51] based on Gene ontology (GO) [52, 53]. A list of highly deleterious genes was subjected to pathway analysis using the Reactome, a curated database [54, 55], and KEGG database. The gene set file was analyzed in an automation mode so that all the genes were in the same line and delimited by commas.

### 2.5 Tissue specific expression of targeted genes in normal and diseased state

**Fig 1:**
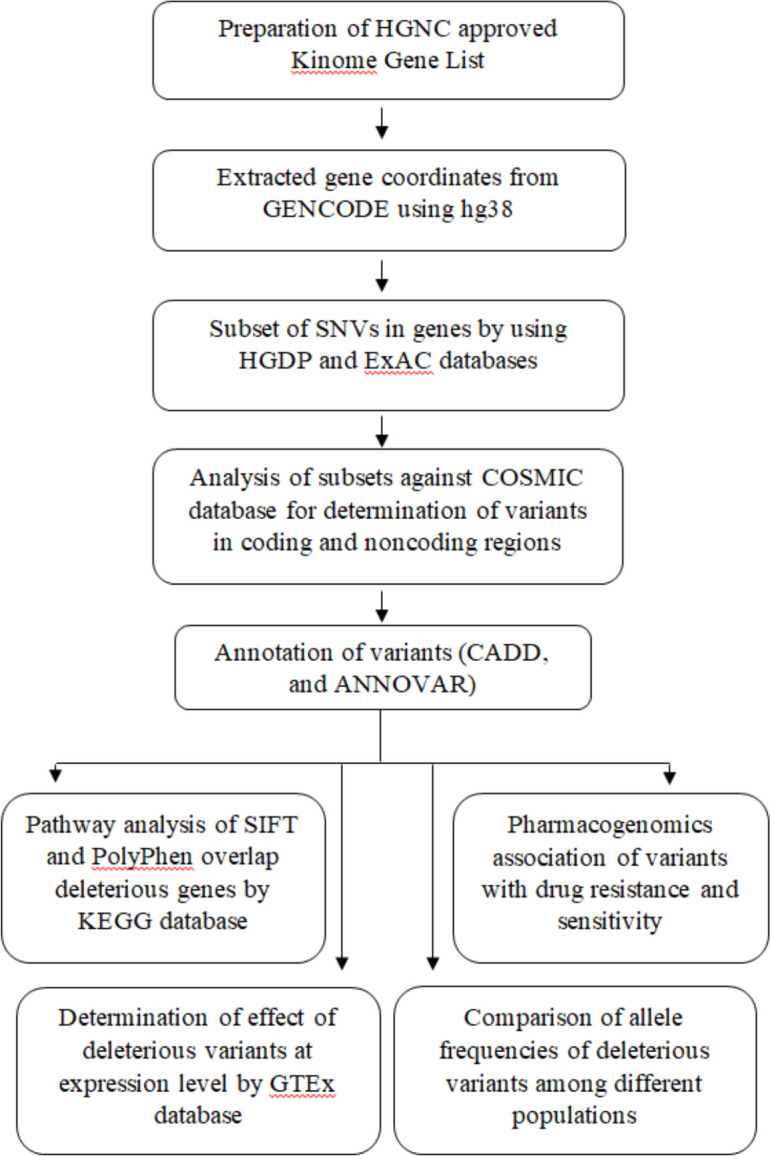
Workflow of the present study for the human Kinome analysis.

To study the functional effects of mutations linked with phenotype in various cancers, we performed a systemized analysis. Here, we selected the most significant genes to determine their expression in normal and diseased conditions, such as, breast ductal carcinoma, non-small cell lung cancer, prostate adenocarcinoma, hepatocellular carcinoma,, and pancreatic ductal carcinoma (top-rated cancer according to incidence and mortality globally) [56]. For this purpose, we used GTEx portal (https://www.gtexportal.org/home/) which is a valuable public resource designed to investigate the regulation and expression of genes specific to particular tissues. The project has collected samples from 53 non-diseased tissue sites across nearly 1000 individuals, primarily for molecular assays including RNA-seq [57]. We also used the Expression Atlas database (http://www.ebi.ac.uk/gxa/home) [58] which provides powerful methods to find information about genes and expression of protein in diverse species, in the respective biological conditions. The workflow of the study has been depicted in **Fig 1.**

### 2.6 Pharmacogenomics of the selected genes

The association of genetic variants with drug sensitivity and resistance mechanisms was also assessed by using the Pharmacogenomics software. To identify the drugs that target the prioritized genes, we first used Connectivity Map from Broad Institute (https://clue.io, data version 1.1.1.2, software version 1.1.1.39) [59]. This resource provides curated datasets generated from testing well-annotated genetic and small molecular perturbations in a variety of cell lines. We exported the compounds and their mechanisms of action. To identify newer drugs and associations that may not be included in the Connectivity Map, we evaluated the Chong et al. (2017) study for drugs that target the prioritized genes in this study (http://clincancerres.aacrjournals.org/content/23/1/204.long).

## 3. Result

### 3.1 Gene ontology

The entire human kinome consisted of 500 genes primarily involved in signal transduction pathways regulating the vital biological processes, such as, cell growth, survival, apoptosis, proliferation, differentiation, metabolic processes, anatomical structure formation, cellular compartment organization and genesis, developmental processes, and organismal processes (S1 Fig).

### 3.2 Kinome annotation, filtration and prioritization

All the SNVs in intronic, exonic, and flanking regions of the kinome genes under study, extracted from the HGDP and ExAC datasets, were analyzed for mutational load by applying our analysis pipeline (Table 1). The total number of single nucleotide substitution sites in the kinome genes was higher in the ExAC database (231,867) than in the HGDP database (9,358). However, there was higher number of per person SNV sites in the HGDP (8.91) than in the ExAC subset (3.82). For assessing the likely association of the subset SNVs with cancers, we further used COSMIC datasets to calculate the proportions of variants in the non-coding and coding regions, as well as the functional consequences such as synonymous, nonsynonymous, frameshift or non-frameshift in coding regions of both the datasets. Higher numbers of the annotated, non-coding, and coding variants was found in the ExAC database (4867, 952, 3915) as compared to the HGDP dataset (121, 61, 60). Notably, a higher non-synonymous/synonymous ratio was observed in HGDP SNVs (4.57) vs the ExAC SNVs (2.59). No stop-gain or stop-loss mutation was found in the HGDP variants, whereas 99 stop-gain and 108 stop-loss variants were observed in the ExAC. The number of frameshift variants was also found higher in ExAC datasets (233) than in the HGDP (15).

**Table 1:** The number of variants within the coordinates of human kinome genes set filtered from the two databases.

Number of Kinome Genes	500	
	HGDP Database	ExAC Database
Total no. of Variants	9,358	231,867
Annotation on the Basis of Cosmic Database		
Annotated Variants	121	4,867
Non-Coding Variants	61	952
Coding Variants	60	3,915
Synonymous	7	511
Non-Synonymous	32	1,323
Stop gained	0	99
Stop loss	0	108
Frameshift	15	233
SIFT_D	21	752
PolyPhen_D	18	653
SIFT_PolyPhen_D_overlap	6	341
SIFT_PolyPhen_D_overlap_CAAD≥20	6	33
SIFT_PolyPhen_D_overlap_CAAD≥20_AF≤0.01	0	33

**Fig 2:**
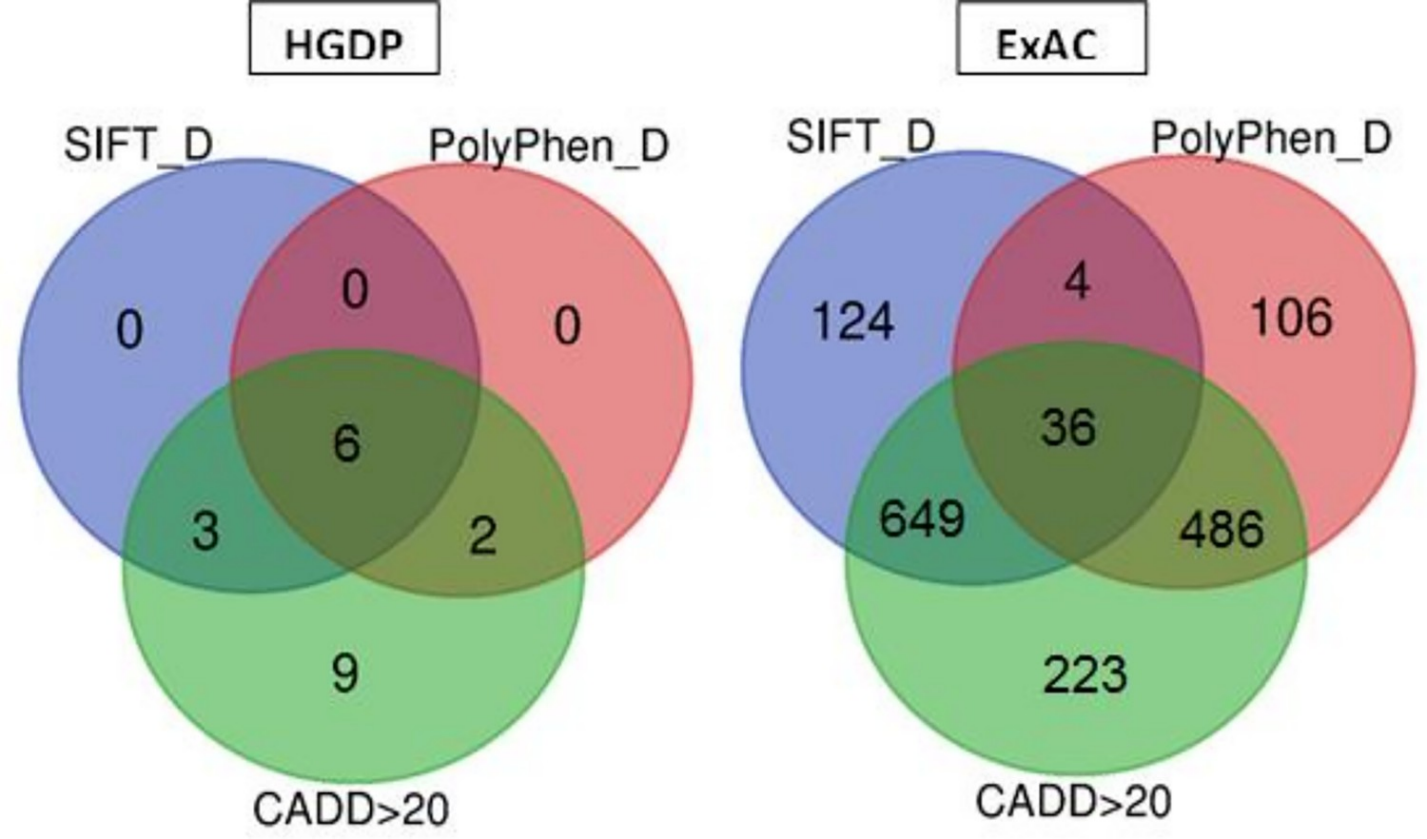
Venn diagram showing the number of variants predicted as deleterious in the human kinome genes by using the three tools (SIFT, PolyPhen, and CADD) in HGDP and ExAC datasets.

**Fig 3:**
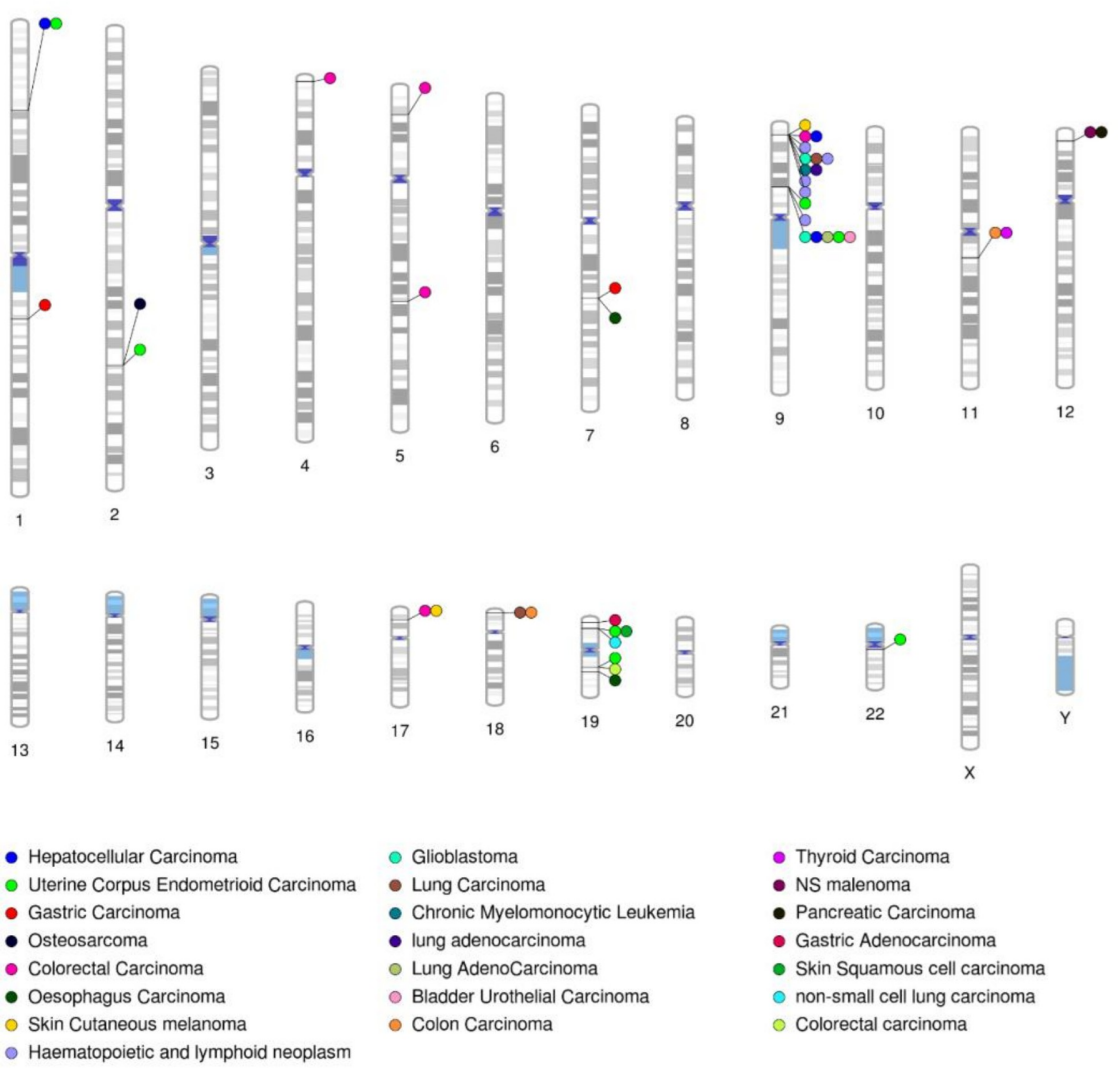
Genomic positions of genes harboring the variants associated with various cancers as filtered from the ClinVar database. One circle beside the chromosomes denotes one gene, and the color indicates type of cancer. The loci on chromosomes 9 and 19 are richer in variants with clinical significance.

A total of 39 and 1,405 variants were predicted to be deleterious by the SIFT and PolyPhen2 tools in the HGDP and ExAC datasets, out of which 6 and 36 variants were found as deleterious by the three in silico tools (SIFT, PolyPhen2, and CADD score≥20) in the HGDP and ExAC datasets There was an overlapping of a few variants between three categories of cancer genes (Fig 2, S1 Table). Population-wise, the Non-Finnish Europeans contained the highest number of deleterious variants (26 SNVs), followed by the Africans (14 SNVs), East Asians (13 SNVs), South Asians (12 SNVs), and Americans (9 SNVs). We highlighted the genomic locations of the genes harboring the deleterious variants associated with different types of cancers with the help of a phenogram (Fig 3). This analysis highlighted the loci of chromosomes 9 and 19 to be rich in deleterious variants involved in different types of cancers.

### 3.3 Functional consequences of deleterious genes by the enrichment analysis

To further understand the function and mechanism of the identified genes, functional and pathway enrichment analyses were performed using the PANTHER database. The GO term enrichment analysis showed that in the biological processes-associated category, the deleterious gene variants were enriched in protein phosphorylation, phosphate-containing compound metabolic process, phosphorus metabolic process, cellular protein modification process and protein modification process (S2 Table). Moreover, for molecular function, the deleterious genes were enriched in protein kinase activity, phosphotransferase activity, alcohol group as acceptor, kinase activity, transferase activity, transferring phosphorus-containing groups, ATP binding, adenyl ribonucleotide binding, drug binding, and catalytic activity, acting on a protein (S3 Table). Furthermore, the PANTHER pathway analysis also showed that deleterious gene variants were significantly enriched in cellular processes, such as, receptor complex, nucleus and nuclear body, transferase complex, transferring phosphorus-containing groups, cytosol, organelle, protein kinase complex, and nucleoplasm part (S4 Table).

To explore the biological pathways affected by the deleterious variants of kinases, we performed a pathway enrichment analysis. The details of pathways associated with variants involved in carcinogenesis are described in S5 Table, and the significant top-ranked pathways affected by the deleterious variants (p<0.05) are shown in Table 2. Following the pathway analysis for the potentially deleterious 23 kinase genes, a total of 422 pathways from the REACTOME dataset were identified as significant (S2 Fig). The strongest association found was between *FGFR3* and *NTRK1* gene variants. Pathways such as signaling by activated point mutants of *FGFR3* (P = 0.000908), *FGFR3* mutant receptor activation (P = 0.001590949), *FGFR3* ligand binding and activation (P = 0.017778563), SHC-mediated cascade: *FGFR3* (P = 0.040249136) were associated with *FGFR3*. Whereas, TRKA activation by *NGF* (P = 0.020523118), activation of TRKA receptors (P = 0.020523118), expression and processing of neurotrophins (P = 0.035821162), *NGF* processing (P = 0.035821162), prolonged *ERK* activation events (P = 0.05928807) were found to be associated with *NTRK1*. The KEGG pathways analysis revealed that *NTRK1* was involved in the MAPK signaling pathway (hsa04010), Ras signaling pathway (hsa04014), calcium signaling pathway (hsa04020), PI3K-Akt signaling pathway (hsa04151), apoptosis (hsa04210), neurotrophin signaling pathway (hsa04722), inflammatory mediator regulation of TRP channels (hsa04750), pathways in cancer (hsa05200), transcriptional misregulation in cancer (hsa05202), and central carbon metabolism in cancer (hsa05230). Likewise, the *FGFR3* was found to be involved in the bladder cancer (hsa05219), MicroRNAs in cancer (hsa05206), EGFR tyrosine kinase inhibitor resistance (hsa01521), MAPK signaling pathway (hsa04010), Ras signaling pathway (hsa04014), Rap1 signaling pathway (hsa04015), calcium signaling pathway (hsa04020), PI3K-Akt signaling pathway (hsa04151), signaling pathways regulating pluripotency of stem cells (hsa04550), pathways in cancer (hsa05200), and central carbon metabolism in cancer (hsa05230).

**Table 2:** The top-ranked pathways (p < 0.05) affected from the deleterious variants.

Pathway Identifier	Pathway name	Entities					Genes
		Observed	Total	Ratio	p-value	FDR	
R-HSA-9006934	Signaling by Receptor Tyrosine Kinases	8	621	0.040901008	9.86E-07	1.29E-04	NTRK1, YES1, AXL, INSR, JAK2, FGFR3
R-HSA-2033514	FGFR3 mutant receptor activation	3	17	0.001119673	1.84E-06	1.29E-04	FGFR3
R-HSA-1839130	Signaling by activated point mutants of FGFR3	3	17	0.001119673	1.84E-06	1.29E-04	FGFR3
R-HSA-5655332	Signaling by FGFR3 in disease	3	33	0.002173484	1.33E-05	6.90E-04	FGFR3
R-HSA-187042	TRKA activation by NGF	2	5	3.29E-04	2.27E-05	9.53E-04	NTRK1
R-HSA-5663202	Diseases of signal transduction by growth factor receptors and second messengers	6	498	0.032799842	4.41E-05	0.001544731	YES1, RPS6KB2, JAK2, FGFR3
R-HSA-187015	Activation of TRKA receptors	2	10	6.59E-04	9.03E-05	0.002710212	NTRK1
R-HSA-74751	Insulin receptor signalling cascade	3	72	0.004742146	1.33E-04	0.003459025	INSR, FGFR3
R-HSA-1226099	Signaling by FGFR in disease	3	82	0.005400777	1.95E-04	0.004480154	FGFR3
R-HSA-5654227	Phospholipase C-mediated cascade; FGFR3	2	18	0.001185536	2.91E-04	0.005733789	FGFR3
R-HSA-190239	FGFR3 ligand binding and activation	2	19	0.0012514	3.24E-04	0.005733789	FGFR3
R-HSA-74752	Signaling by Insulin receptor	3	99	0.006520451	3.38E-04	0.005733789	INSR, FGFR3
R-HSA-169893	Prolonged ERK activation events	2	20	0.001317263	3.58E-04	0.005733789	NTRK1
R-HSA-5654710	PI-3K cascade:FGFR3	2	24	0.001580715	5.14E-04	0.006977232	FGFR3
R-HSA-5654704	SHC-mediated cascade:FGFR3	2	26	0.001712442	6.03E-04	0.006977232	FGFR3
R-HSA-2219528	PI3K/AKT Signaling in Cancer	3	124	0.008167029	6.49E-04	0.006977232	RPS6KB2, FGFR3
R-HSA-5654706	FRS-mediated FGFR3 signaling	2	27	0.001778305	6.49E-04	0.006977232	FGFR3
R-HSA-9670439	Signaling by phosphorylated juxtamembrane, extracellular and kinase domain KIT mutants	2	28	0.001844168	6.98E-04	0.006977232	YES1, JAK2
R-HSA-6804115	TP53 regulates transcription of additional cell cycle genes whose exact role in the p53 pathway remain uncertain	2	28	0.001844168	6.98E-04	0.006977232	PLK2
R-HSA-9669938	Signaling by KIT in disease	2	28	0.001844168	6.98E-04	0.006977232	YES1, JAK2
R-HSA-6811558	PI5P, PP2A and IER3 Regulate PI3K/AKT Signaling	3	129	0.008496345	7.27E-04	0.007273749	INSR, FGFR3
R-HSA-199418	Negative regulation of the PI3K/AKT network	3	137	0.00902325	8.65E-04	0.007456964	INSR, FGFR3
R-HSA-1257604	PIP3 activates AKT signaling	4	321	0.021142067	8.96E-04	0.007456964	INSR, RPS6KB2, FGFR3
R-HSA-162582	Signal Transduction	11	3023	0.199104261	9.32E-04	0.007456964	NTRK1, YES1, TRIO, AXL, INSR, RPS6KB2, JAK2, FGFR3
R-HSA-5654732	Negative regulation of FGFR3 signaling	2	34	0.002239347	0.001023658	0.008189261	FGFR3

### 3.4 Comparison of NTRK1 and FGFR3 gene expression in normal and cancerous tissues

**Fig 4:**
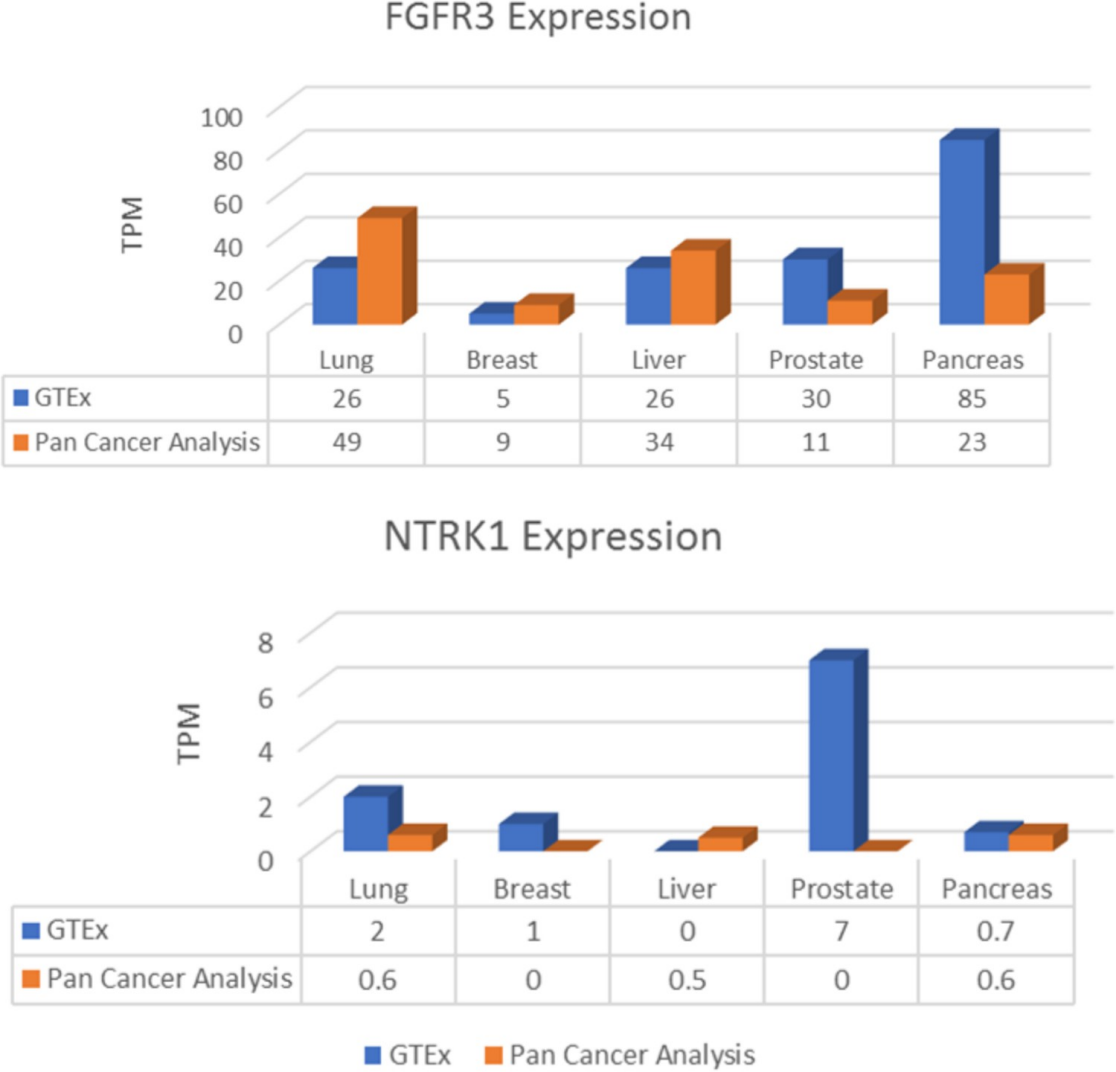
Graphical representation of TPM values of highly deleterious kinase genes (NTRK1 and FGFR3) involved in various cancers. The TPM values were retrieved from the Pan Cancer Analysis of Whole Genome Project compared with normal cells of the respective organs in the GTEx database.

In the present study, the expression of NTRK1 in various cancers and their related normal tissues was performed through the Expression Atlas. The transcripts per million reads (TPM) level of NTRK1 was found to be lower in the lung, breast, prostate and pancreas cancers when compared to their normal state. However, an increased TPM of NTRK1 was found in liver cancer when compared to normal liver tissue (Fig 4). We evaluated the expression levels of FGFR3 in various fetal cancers and their related normal tissues through the Expression Atlas. According to the analysis, a higher expression of *FGFR3* was recorded in the lung, breast, and liver cancers, whereas its expression was significantly decreased in prostate and pancreas cancerous states compared with the respective normal tissues (Fig 4). The Expression Atlas analysis indicates the expression dependent activity of both NTRK1 and FGFR3 in various organs under physiologically normal and cancerous states.

### 3.5 Pharmacogenomics of NKTR1 and FGFR3

This analysis validated some of the drugs extracted from Connectivity Map and provided additional drugs that might be effective in the cancer treatment. We combined drugs from both Connectivity Map and Chong et al. study to generate a final list of drugs that target NTRK1 and FGFR3 (Tables 3 & 4). There were 13 drugs that target the NTRK1, and 6 drugs which target the FGFR3 protein.

**Table 3:** List of drugs that target NTRK1.

Name	Target	Mechanism Of Action
GW-5074	NTRK1	Leucine rich repeat kinase inhibitor, RAF inhibitor
AG-879	NTRK1	Angiogenesis inhibitor, Tyrosine kinase inhibitor, VEGFR inhibitor
Amitriptyline	NTRK1	Norepinephrine inhibitor, Norepinephrine reuptake inhibitor, Serotonin receptor antagonist, Serotonin reuptake inhibitor
GW-441756	NTRK1	Growth factor receptor inhibitor.
Imatinib	NTRK1	BCR-ABL kinase inhibitor, KIT inhibitor, PDGFR receptor inhibitor
Danusertib	NTRK1	Aurora kinase inhibitor, Growth factor receptor inhibitor
Lestaurtinib	NTRK1	FLT3 inhibitor, Growth factor receptor inhibitor, JAK inhibitor
Larotrectinib	NTRK gene fusion	TrkA, TrkB, TrkC inhibitor
Crizotinib	NTRK	LK, CYP2B6, CYP3A5, MET, MST1R, ROS2
Entrectinib	NTRK	TrkA-, TrkB-, TrkC-, ROS1- and ALK
AZD-7451	NTRK	TrK inhibitor
Dovitinib	NTRK	EGFR inhibitor, FLT3 inhibitor, FGFR inhibitor, PDGFR receptor inhibitor, VEGFR inhibitor

**Table 4:** List of drugs that target FGFR3.

Name	Target	Mechanism of Action
PD-173074	FGFR3	FGFR inhibitor, VEGFR inhibitor
Dovitinib	FGFR3	EGFR inhibitor, FLT3 inhibitor, FGFR inhibitor, PDGFR receptor inhibitor, VEGFR inhibitor
Pazopanib	FGFR3	KIT inhibitor, PDGFR receptor inhibitor, VEGFR inhibitor
Masitinib	FGFR3	KIT inhibitor, PDGFR receptor inhibitor, SRC inhibitor
Brivanib	FGFR3	FGFR inhibitor, VEGFR inhibitor
ENMD-2076	FGFR3	FLT3 inhibitor, VEGFR inhibitor, Aurora kinase inhibitor

## 4. Discussion

The predisposing pathogenic mutations in the humans enhance the susceptibility to the associated diseases. Here we prioritized potential pathogenic mutations in the global populations by using the publicly available database of human genetic variations (ExAC, and HGDP) by employing different *in silico* tools which predict the effect of genetic variations. We determined the deleterious mutations in the genes of kinase category already associated with different cancers. To the best of our knowledge this is the first report to present the predisposing potential deleterious mutations in the kinome genes.

The presence of large number of predisposing detrimental variations can impact the overall fitness of a population [60, 61]. In this study, the number of deleterious variants in the kinome genes were found higher in non-Finnish Europeans, and Africans compared with other populations of the world. This finding correlates with the previous reports where a significant percentage of uncommon variations in populations from Europe and Africa were deleterious. Also, there was a marginal difference in the number of rare frequency deleterious variants (derived allele frequency (DAF) < 1%) in the European-Americans and African-Americans [62]. One possible explanation for the relatively higher percentage of high-frequency detrimental variations is due to the well-known bottleneck that the European populations went through, which may have caused harmful mutations to drift to high frequencies. Also, it has been shown in a recent study that an individual’s deleterious genetic variations in cancer predisposing genes-sets are linked to higher risk, earlier onset age, elevated M1 macrophages in tumors, and higher tumor mutational burden in particular malignancies [63].

Through the genes set enrichment analysis, we prioritized two genes *NTRK1* and *FGFR3* having the most significant interactions. *NTRK1* gene encodes for a protein known as tropomyosin receptor kinase A (TRKA) involved in various processes, especially in the development and survival of nerve cells. Its altered expression has been reported to result in pathogenesis such as cancers. Previously, a more aggressive subset of soft tissue tumors with distinctive LPF-like morphology were found associated with NTRK1-associated genetic abnormalities [64]. In other reports, NTRK1 rearrangements in metastatic gastrointestinal cancer, colorectal cancer, glioblastoma, and non-small cell lung cancer patients were observed [65, 66]. This rearrangement might be associated with altered expression of the gene. Herein, the lower gene expression of *NTRK1* in the cancer tissues might indicate suppression of its associated pathways leading to cancer.

The *FGFR3* gene encodes a protein known as fibroblast growth factor receptor 3. Its expression also varies between normal and diseased conditions such as cancer. The *FGFR3* seems to have diverse roles as its expression is upregulated in some cancers and downregulated in others. Its activation has previously been reported in bladder and skin cancers [67]. In the bladder cancer, the FGFR3 is considered as an actionable target [68]. In triple negative breast cancer, aberrant FGFR3 activation was identified through the mass spectrometry (MS)-based tyrosine phosphorylation profiling [69]. Further, reduced sensitivity to tamoxifen (an estrogen blocker) was observed in vitro in the michigan cancer foundation—1 cells (MCF7) with FGF1. From the same study, the tissue microarray expression data of tamoxifen resistant breast tumors demonstrated that FGFR3 expression was significantly increased compared with the normal cells, resulting in activation of the mitogen-activated protein kinase (MAPK) and phosphoinositide 3-kinase (PI3K) signalling pathways, both of which have been implicated in tamoxifen resistance in breast cancer [70]. These evidences suggest convincing role of *FGFR3* in cancers predispositions and disease outcome.

## 5. Conclusion

The study, for the first time, estimates the predisposing mutations in the coding regions of the human kinases genes in the global populations by using the HGDP, and ExAC databases. The analysis highlighted that the Non-Finnish Europeans contained the highest number of deleterious SNVs (26), followed by Africans (14 SNVs), East Asians (13 SNVs), and South Asians (12 SNVs). The identification of deleterious mutations enabled exploration of the underlying mechanisms potentially involved in the onset of various sporadic cancers in the global populations. The gene set enrichment analysis highlighted the likely role of NTRK1 and FGFR3 proteins in sporadic carcinogenesis. The pathways identified in this analysis indicate the underlying molecular mechanisms which might be instrumental in managing the cancers without a known causative exposure. The identified drug targets may be used in personalized cancer management. The analysis based on the bioinformatics and in-silico tools is the limiting factor, and the findings can further be validated in the cell lines or animal models. In future, the identified variants can be screened in the patients to find the penetrance of variants in the populations.

## Supporting information

S1 Fig Gene ontology of the kinome genes. Majority of the genes are involved in different cellular processes, biological regulation, metabolic processes, response to stimulus, and signaling.
(DOCX)

S2 Fig Different pathways and the number of their associated genes of current analysis. (DOCX)

S1 Table Kinome annotation, filtration and prioritization. (XLSX)

S2 Table Showing biological processes affected by the deleterious variants by panther. (DOCX)

S3 Table Showing molecular functions affected by the deleterious variants by panther. (DOCX)

S4 Table Cellular components affected by the deleterious variants (by panther database). (DOCX)

S5 Table The total number of pathways associated with variants involved in carcinogenesis. (DOCX)

S1 File Request for change to authorship. (DOCX)
