# Supplementary material for: Predisposing deleterious variants in the cancer-associated human kinases in the global populations
Source: PLoS One. 2024 Apr 18;19(4):e0298747. doi: 10.1371/journal.pone.0298747 (PMC11025791; doi:10.1371/journal.pone.0298747)
Supplement: S1 Fig — Majority of the genes are involved in different cellular processes, biological regulation, metabolic processes, response to stimulus, and signaling. (DOCX) [file pone.0298747.s001.docx]

**
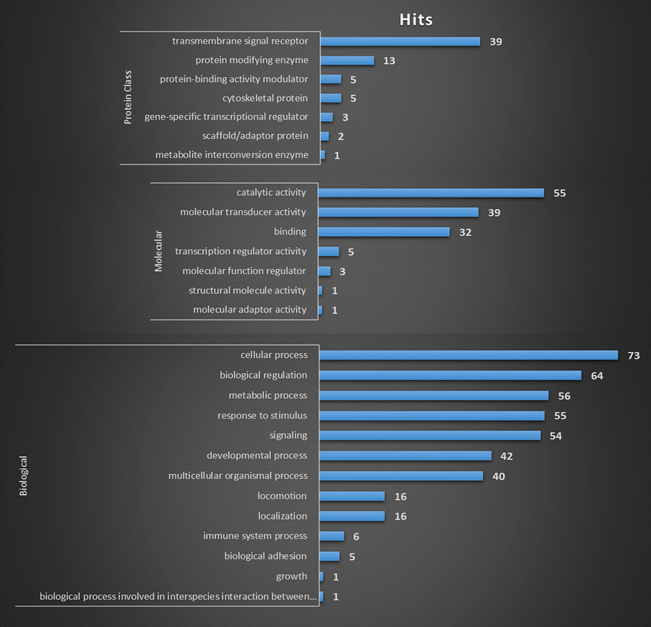
**

**Supplementary Figure S1:** Gene ontology of the kinome genes. In biological processes, majority of the genes are involved in different cellular processes, biological regulation, metabolic processes, response to stimulus, and signaling. In molecular functions, majority of genes were found to be involved in catalytic activity, and molecular transfer activity. In the proteins classes, transmembrane signal receptors are far more than other classes.
