# Supplementary material for: Predisposing deleterious variants in the cancer-associated human kinases in the global populations
Source: PLoS One. 2024 Apr 18;19(4):e0298747. doi: 10.1371/journal.pone.0298747 (PMC11025791; doi:10.1371/journal.pone.0298747)
Supplement: S2 Fig — (DOCX) [file pone.0298747.s002.docx]

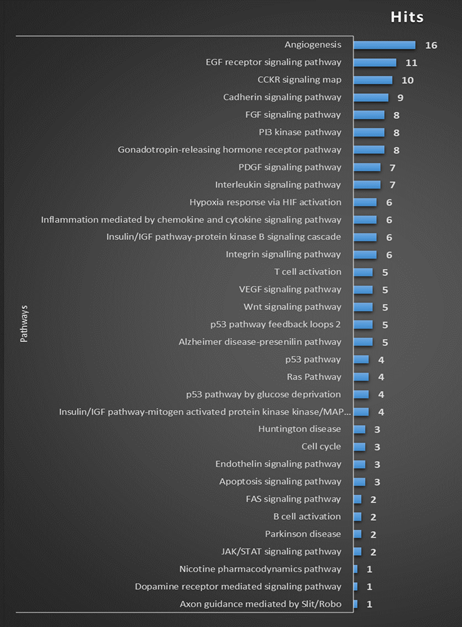


**Supplementary Figure S2:** Different pathways and the number of their associated genes of current analysis. The pathway of angiogenesis, EGF receptor signaling pathway, CCKR signaling map, cadherin signaling pathway, and FGF signaling pathway were found more enriched than others.
