# Supplementary material for: Predisposing deleterious variants in the cancer-associated human kinases in the global populations
Source: PLoS One. 2024 Apr 18;19(4):e0298747. doi: 10.1371/journal.pone.0298747 (PMC11025791; doi:10.1371/journal.pone.0298747)
Supplement: S2 Table — (DOCX) [file pone.0298747.s004.docx]

**Supplementary Table S2:** Various biological processes affected by the deleterious variants, analyzed by the online Panther database.

| **Biological process / GO ID** | **Count** | **%** | **Upload_1 (P-value)** |
| --- | --- | --- | --- |
| protein phosphorylation (GO:0006468) | 83 | 90.2173913 | 1.06E-95 |
| phosphorylation (GO:0016310) | 84 | 91.30434783 | 3.12E-87 |
| phosphate-containing compound metabolic process (GO:0006796) | 84 | 91.30434783 | 5.44E-69 |
| phosphorus metabolic process (GO:0006793) | 84 | 91.30434783 | 1.51E-68 |
| cellular protein modification process (GO:0006464) | 86 | 93.47826087 | 5.53E-60 |
| protein modification process (GO:0036211) | 86 | 93.47826087 | 5.53E-60 |
| macromolecule modification (GO:0043412) | 86 | 93.47826087 | 1.35E-57 |
| **Biological process / GO ID** | **Count** | **%** | **Upload_1 (P-value)** |
| cellular protein metabolic process (GO:0044267) | 86 | 93.47826087 | 3.07E-53 |
| peptidyl-amino acid modification (GO:0018193) | 58 | 63.04347826 | 1.32E-51 |
| protein autophosphorylation (GO:0046777) | 39 | 42.39130435 | 5.95E-49 |
| protein metabolic process (GO:0019538) | 86 | 93.47826087 | 4.62E-47 |
| cellular macromolecule metabolic process (GO:0044260) | 87 | 94.56521739 | 1.49E-43 |
| organonitrogen compound metabolic process (GO:1901564) | 86 | 93.47826087 | 7.18E-39 |
| peptidyl-tyrosine phosphorylation (GO:0018108) | 33 | 35.86956522 | 3.66E-38 |
| peptidyl-tyrosine modification (GO:0018212) | 33 | 35.86956522 | 5.82E-38 |
| macromolecule metabolic process (GO:0043170) | 88 | 95.65217391 | 2.57E-37 |
| nitrogen compound metabolic process (GO:0006807) | 88 | 95.65217391 | 3.65E-32 |
| intracellular signal transduction (GO:0035556) | 53 | 57.60869565 | 4.08E-30 |
| cellular metabolic process (GO:0044237) | 88 | 95.65217391 | 5.71E-30 |
| primary metabolic process (GO:0044238) | 88 | 95.65217391 | 1.12E-29 |
| organic substance metabolic process (GO:0071704) | 88 | 95.65217391 | 4.05E-28 |
| metabolic process (GO:0008152) | 88 | 95.65217391 | 4.44E-26 |
| peptidyl-serine phosphorylation (GO:0018105) | 23 | 25 | 1.68E-23 |
| peptidyl-serine modification (GO:0018209) | 23 | 25 | 3.05E-22 |
| signal transduction (GO:0007165) | 68 | 73.91304348 | 1.57E-20 |
| signaling (GO:0023052) | 68 | 73.91304348 | 7.00E-19 |
| cell communication (GO:0007154) | 68 | 73.91304348 | 2.72E-18 |
| regulation of intracellular signal transduction (GO:1902531) | 42 | 45.65217391 | 1.55E-16 |
| **Biological process / GO ID** | **Count** | **%** | **Upload_1 (P-value)** |
| enzyme linked receptor protein signaling pathway (GO:0007167) | 29 | 31.52173913 | 1.68E-16 |
| cellular response to stimulus (GO:0051716) | 71 | 77.17391304 | 7.59E-16 |
| trans membrane receptor protein tyrosine kinase signaling pathway (GO:0007169) | 25 | 27.17391304 | 1.40E-15 |
| signal transduction by protein phosphorylation (GO:0023014) | 22 | 23.91304348 | 3.18E-15 |
| positive regulation of kinase activity (GO:0033674) | 25 | 27.17391304 | 2.62E-14 |
| regulation of kinase activity (GO:0043549) | 29 | 31.52173913 | 2.90E-14 |
| regulation of signal transduction (GO:0009966) | 49 | 53.26086957 | 1.63E-13 |
| positive regulation of transferase activity (GO:0051347) | 25 | 27.17391304 | 4.26E-13 |
| positive regulation of phosphorylation (GO:0042327) | 30 | 32.60869565 | 4.47E-13 |
| regulation of transferase activity (GO:0051338) | 29 | 31.52173913 | 7.07E-13 |
| positive regulation of intracellular signal transduction (GO:1902533) | 30 | 32.60869565 | 8.22E-13 |
| positive regulation of cellular process (GO:0048522) | 61 | 66.30434783 | 1.08E-12 |
| positive regulation of protein phosphorylation (GO:0001934) | 29 | 31.52173913 | 1.11E-12 |
| positive regulation of cellular protein metabolic process (GO:0032270) | 35 | 38.04347826 | 1.18E-12 |
| positive regulation of signal transduction (GO:0009967) | 35 | 38.04347826 | 2.06E-12 |
| regulation of cell communication (GO:0010646) | 50 | 54.34782609 | 2.22E-12 |
| positive regulation of phosphorus metabolic process (GO:0010562) | 30 | 32.60869565 | 2.66E-12 |
| positive regulation of phosphate metabolic process (GO:0045937) | 30 | 32.60869565 | 2.66E-12 |
| regulation of signaling (GO:0023051) | 50 | 54.34782609 | 3.08E-12 |
| **Biological process / GO ID** | **Count** | **%** | **Upload_1 (P-value)** |
| regulation of protein kinase activity (GO:0045859) | 26 | 28.26086957 | 3.14E-12 |
| positive regulation of protein metabolic process (GO:0051247) | 35 | 38.04347826 | 8.00E-12 |
| positive regulation of protein kinase activity (GO:0045860) | 22 | 23.91304348 | 9.56E-12 |
| positive regulation of protein modification process (GO:0031401) | 30 | 32.60869565 | 2.26E-11 |
| positive regulation of biological process (GO:0048518) | 63 | 68.47826087 | 2.34E-11 |
| regulation of cellular process (GO:0050794) | 83 | 90.2173913 | 2.47E-11 |
| regulation of protein phosphorylation (GO:0001932) | 32 | 34.7826087 | 2.59E-11 |
| regulation of phosphorylation (GO:0042325) | 33 | 35.86956522 | 3.26E-11 |
| regulation of response to stimulus (GO:0048583) | 53 | 57.60869565 | 3.36E-11 |
| positive regulation of cell communication (GO:0010647) | 35 | 38.04347826 | 3.39E-11 |
| positive regulation of signaling (GO:0023056) | 35 | 38.04347826 | 3.88E-11 |
| activation of protein kinase activity (GO:0032147) | 18 | 19.56521739 | 4.72E-11 |
| positive regulation of molecular function (GO:0044093) | 35 | 38.04347826 | 5.18E-11 |
| cell surface receptor signaling pathway (GO:0007166) | 40 | 43.47826087 | 6.09E-11 |
| MAPK cascade (GO:0000165) | 18 | 19.56521739 | 7.10E-11 |
| response to stimulus (GO:0050896) | 73 | 79.34782609 | 9.19E-11 |
| regulation of protein metabolic process (GO:0051246) | 42 | 45.65217391 | 1.98E-10 |
| regulation of biological process (GO:0050789) | 84 | 91.30434783 | 3.63E-10 |
| regulation of cellular protein metabolic process (GO:0032268) | 40 | 43.47826087 | 6.53E-10 |
| positive regulation of macromolecule metabolic process (GO:0010604) | 45 | 48.91304348 | 7.08E-10 |
|  |  |  |  |
| **Biological process / GO ID** | **Count** | **%** | **Upload_1 (P-value)** |
| positive regulation of response to stimulus (GO:0048584) | 38 | 41.30434783 | 9.65E-10 |
| regulation of phosphate metabolic process (GO:0019220) | 33 | 35.86956522 | 1.01E-09 |
| peptidyl-tyrosine autophosphorylation (GO:0038083) | 9 | 9.782608696 | 1.02E-09 |
| regulation of phosphorus metabolic process (GO:0051174) | 33 | 35.86956522 | 1.04E-09 |
| positive regulation of metabolic process (GO:0009893) | 46 | 50 | 2.39E-09 |
| positive regulation of cellular metabolic process (GO:0031325) | 44 | 47.82608696 | 2.78E-09 |
| regulation of protein modification process (GO:0031399) | 33 | 35.86956522 | 3.53E-09 |
| positive regulation of nitrogen compound metabolic process (GO:0051173) | 43 | 46.73913043 | 3.85E-09 |
| positive regulation of MAPK cascade (GO:0043410) | 19 | 20.65217391 | 2.16E-08 |
| biological regulation (GO:0065007) | 84 | 91.30434783 | 2.25E-08 |
| regulation of primary metabolic process (GO:0080090) | 58 | 63.04347826 | 6.10E-08 |
| regulation of MAPK cascade (GO:0043408) | 21 | 22.82608696 | 8.06E-08 |
| cellular process (GO:0009987) | 89 | 96.73913043 | 8.35E-08 |
| regulation of nitrogen compound metabolic process (GO:0051171) | 57 | 61.95652174 | 9.05E-08 |
| regulation of macromolecule metabolic process (GO:0060255) | 58 | 63.04347826 | 9.58E-08 |
| regulation of cellular metabolic process (GO:0031323) | 58 | 63.04347826 | 2.36E-07 |
| positive regulation of catalytic activity (GO:0043085) | 27 | 29.34782609 | 4.01E-07 |
| stress-activated protein kinase signaling cascade (GO:0031098) | 11 | 11.95652174 | 6.50E-07 |
|  |  |  |  |
| **Biological process / GO ID** | **Count** | **%** | **Upload_1 (P-value)** |
| regulation of metabolic process (GO:0019222) | 59 | 64.13043478 | 9.26E-07 |
| regulation of molecular function (GO:0065009) | 40 | 43.47826087 | 1.08E-06 |
| ematopoietic or lymphoid organ development (GO:0048534) | 18 | 19.56521739 | 1.12E-06 |
| response to stress (GO:0006950) | 41 | 44.56521739 | 1.59E-06 |
| regulation of protein kinase B signaling (GO:0051896) | 12 | 13.04347826 | 2.13E-06 |
| regulation of programmed cell death (GO:0043067) | 27 | 29.34782609 | 2.20E-06 |
| positive regulation of protein kinase B signaling (GO:0051897) | 11 | 11.95652174 | 2.26E-06 |
| hemopoiesis (GO:0030097) | 17 | 18.47826087 | 2.61E-06 |
| immune system development (GO:0002520) | 18 | 19.56521739 | 2.74E-06 |
| cellular developmental process (GO:0048869) | 42 | 45.65217391 | 5.07E-06 |
| regulation of protein serine/threonine kinase activity (GO:0071900) | 16 | 17.39130435 | 6.67E-06 |
| cellular response to stress (GO:0033554) | 27 | 29.34782609 | 7.06E-06 |
| cell differentiation (GO:0030154) | 41 | 44.56521739 | 9.13E-06 |
| cellular component organization or biogenesis (GO:0071840) | 53 | 57.60869565 | 9.86E-06 |
| regulation of cell death (GO:0010941) | 27 | 29.34782609 | 1.17E-05 |
| cellular component organization (GO:0016043) | 52 | 56.52173913 | 1.19E-05 |
| regulation of catalytic activity (GO:0050790) | 32 | 34.7826087 | 1.26E-05 |
| multicellular organismal process (GO:0032501) | 58 | 63.04347826 | 1.87E-05 |
| positive regulation of protein serine/threonine kinase activity (GO:0071902) | 13 | 14.13043478 | 2.66E-05 |
| positive regulation of cell proliferation (GO:0008284) | 20 | 21.73913043 | 2.87E-05 |
| regulation of apoptotic process (GO:0042981) | 25 | 27.17391304 | 4.67E-05 |
|  |  |  |  |
| **Biological process / GO ID** | **Count** | **%** | **Upload_1 (P-value)** |
| positive regulation of phosphatidylinositol 3-kinase signaling (GO:0014068) | 8 | 8.695652174 | 5.70E-05 |
| histone phosphorylation (GO:0016572) | 6 | 6.52173913 | 7.38E-05 |
| developmental process (GO:0032502) | 51 | 55.43478261 | 7.85E-05 |
| cellular component morphogenesis (GO:0032989) | 18 | 19.56521739 | 9.40E-05 |
| negative regulation of programmed cell death (GO:0043069) | 19 | 20.65217391 | 1.05E-04 |
| negative regulation of biological process (GO:0048519) | 48 | 52.17391304 | 1.16E-04 |
| regulation of response to stress (GO:0080134) | 23 | 25 | 1.19E-04 |
| positive regulation of MAP kinase activity (GO:0043406) | 11 | 11.95652174 | 2.18E-04 |
| regulation of MAP kinase activity (GO:0043405) | 12 | 13.04347826 | 3.00E-04 |
| regulation of cellular response to stress (GO:0080135) | 16 | 17.39130435 | 3.14E-04 |
| regulation of developmental process (GO:0050793) | 31 | 33.69565217 | 3.20E-04 |
| anatomical structure development (GO:0048856) | 48 | 52.17391304 | 3.80E-04 |
| negative regulation of cell death (GO:0060548) | 19 | 20.65217391 | 4.11E-04 |
| negative regulation of apoptotic process (GO:0043066) | 18 | 19.56521739 | 4.69E-04 |
| apoptotic signaling pathway (GO:0097190) | 11 | 11.95652174 | 5.05E-04 |
| cell morphogenesis (GO:0000902) | 16 | 17.39130435 | 5.57E-04 |
| phosphatidylinositol phosphorylation (GO:0046854) | 8 | 8.695652174 | 6.40E-04 |
| regulation of phosphatidylinositol 3-kinase signaling (GO:0014066) | 8 | 8.695652174 | 7.25E-04 |
| regulation of cell cycle (GO:0051726) | 20 | 21.73913043 | 8.33E-04 |
| phosphatidylinositol metabolic process (GO:0046488) | 10 | 10.86956522 | 8.90E-04 |
| **Biological process / GO ID** | **Count** | **%** | **Upload_1 (P-value)** |
| ephrin receptor signaling pathway (GO:0048013) | 7 | 7.608695652 | 9.02E-04 |
| cell morphogenesis involved in differentiation (GO:0000904) | 14 | 15.2173913 | 9.46E-04 |
| cellular response to reactive oxygen species (GO:0034614) | 8 | 8.695652174 | 1.10E-03 |
| regulation of cellular component organization (GO:0051128) | 30 | 32.60869565 | 1.20E-03 |
| lipid phosphorylation (GO:0046834) | 8 | 8.695652174 | 1.46E-03 |
| multicellular organism development (GO:0007275) | 45 | 48.91304348 | 1.50E-03 |
| positive regulation of peptidyl-tyrosine phosphorylation (GO:0050731) | 9 | 9.782608696 | 1.58E-03 |
| regulation of multicellular organismal process (GO:0051239) | 33 | 35.86956522 | 1.60E-03 |
| positive regulation of gene expression (GO:0010628) | 26 | 28.26086957 | 1.64E-03 |
| response to reactive oxygen species (GO:0000302) | 9 | 9.782608696 | 1.65E-03 |
| anatomical structure morphogenesis (GO:0009653) | 27 | 29.34782609 | 1.74E-03 |
| axon development (GO:0061564) | 12 | 13.04347826 | 1.80E-03 |
| response to chemical (GO:0042221) | 41 | 44.56521739 | 1.96E-03 |
| regulation of cell adhesion (GO:0030155) | 15 | 16.30434783 | 2.06E-03 |
| regulation of cell motility (GO:2000145) | 17 | 18.47826087 | 2.37E-03 |
| positive regulation of cellular component organization (GO:0051130) | 20 | 21.73913043 | 2.61E-03 |
| regulation of cell proliferation (GO:0042127) | 23 | 25 | 2.66E-03 |
| organelle organization (GO:0006996) | 35 | 38.04347826 | 3.00E-03 |
| regulation of gene expression (GO:0010468) | 41 | 44.56521739 | 3.70E-03 |
| apoptotic process (GO:0006915) | 17 | 18.47826087 | 3.71E-03 |
| positive regulation of organelle organization (GO:0010638) | 14 | 15.2173913 | 3.88E-03 |
| **Biological process / GO ID** | **Count** | **%** | **Upload_1 (P-value)** |
| programmed cell death (GO:0012501) | 18 | 19.56521739 | 5.00E-03 |
| regulation of localization (GO:0032879) | 30 | 32.60869565 | 5.44E-03 |
| system development (GO:0048731) | 40 | 43.47826087 | 7.36E-03 |
| regulation of locomotion (GO:0040012) | 17 | 18.47826087 | 7.39E-03 |
| regulation of cellular component movement (GO:0051270) | 17 | 18.47826087 | 7.94E-03 |
| cell death (GO:0008219) | 18 | 19.56521739 | 8.01E-03 |
| regulation of cell-matrix adhesion (GO:0001952) | 7 | 7.608695652 | 8.65E-03 |
| cellular response to oxidative stress (GO:0034599) | 9 | 9.782608696 | 8.73E-03 |
| positive regulation of macromolecule biosynthetic process (GO:0010557) | 24 | 26.08695652 | 1.04E-02 |
| neurogenesis (GO:0022008) | 22 | 23.91304348 | 1.08E-02 |
| neuron projection morphogenesis (GO:0048812) | 12 | 13.04347826 | 1.09E-02 |
| cell development (GO:0048468) | 22 | 23.91304348 | 1.09E-02 |
| positive regulation of developmental process (GO:0051094) | 20 | 21.73913043 | 1.10E-02 |
| plasma membrane bounded cell projection morphogenesis (GO:0120039) | 12 | 13.04347826 | 1.19E-02 |
| negative regulation of cellular process (GO:0048523) | 41 | 44.56521739 | 1.21E-02 |
| cell projection morphogenesis (GO:0048858) | 12 | 13.04347826 | 1.22E-02 |
| substrate adhesion-dependent cell spreading (GO:0034446) | 5 | 5.434782609 | 1.30E-02 |
| positive regulation of cell migration (GO:0030335) | 12 | 13.04347826 | 1.30E-02 |
| positive regulation of cell death (GO:0010942) | 14 | 15.2173913 | 1.34E-02 |
| response to oxygen-containing compound (GO:1901700) | 21 | 22.82608696 | 1.50E-02 |
| regulation of peptidyl-tyrosine phosphorylation (GO:0050730) | 9 | 9.782608696 | 1.54E-02 |
| generation of neurons (GO:0048699) | 21 | 22.82608696 | 1.54E-02 |
| **Biological process / GO ID** | **Count** | **%** | **Upload_1 (P-value)** |
| leukocyte differentiation (GO:0002521) | 10 | 10.86956522 | 1.65E-02 |
| immune response-activating signal transduction (GO:0002757) | 12 | 13.04347826 | 1.70E-02 |
| positive regulation of cell motility (GO:2000147) | 12 | 13.04347826 | 2.00E-02 |
| regulation of signal transduction by p53 class mediator (GO:1901796) | 7 | 7.608695652 | 2.09E-02 |
| regulation of organelle organization (GO:0033043) | 19 | 20.65217391 | 2.09E-02 |
| regulation of cellular biosynthetic process (GO:0031326) | 38 | 41.30434783 | 2.09E-02 |
| negative regulation of cell communication (GO:0010648) | 19 | 20.65217391 | 2.21E-02 |
| negative regulation of response to stimulus (GO:0048585) | 21 | 22.82608696 | 2.27E-02 |
| negative regulation of signaling (GO:0023057) | 19 | 20.65217391 | 2.31E-02 |
| cell morphogenesis involved in neuron differentiation (GO:0048667) | 11 | 11.95652174 | 2.33E-02 |
| positive regulation of cellular biosynthetic process (GO:0031328) | 24 | 26.08695652 | 2.46E-02 |
| regulation of cell migration (GO:0030334) | 15 | 16.30434783 | 2.56E-02 |
| positive regulation of cellular component movement (GO:0051272) | 12 | 13.04347826 | 2.59E-02 |
| lipid modification (GO:0030258) | 9 | 9.782608696 | 2.61E-02 |
| positive regulation of multicellular organismal process (GO:0051240) | 22 | 23.91304348 | 2.69E-02 |
| response to acid chemical (GO:0001101) | 10 | 10.86956522 | 2.71E-02 |
| positive regulation of epithelial cell migration (GO:0010634) | 7 | 7.608695652 | 2.78E-02 |
| erythrocyte homeostasis (GO:0034101) | 6 | 6.52173913 | 2.83E-02 |
| **Biological process / GO ID** | **Count** | **%** | **Upload_1 (P-value)** |
| negative regulation of signal transduction (GO:0009968) | 18 | 19.56521739 | 3.06E-02 |
| positive regulation of biosynthetic process (GO:0009891) | 24 | 26.08695652 | 3.25E-02 |
| immune response-regulating signaling pathway (GO:0002764) | 12 | 13.04347826 | 3.39E-02 |
| response to organic substance (GO:0010033) | 30 | 32.60869565 | 3.59E-02 |
| positive regulation of locomotion (GO:0040017) | 12 | 13.04347826 | 3.79E-02 |
| positive regulation of ERK1 and ERK2 cascade (GO:0070374) | 8 | 8.695652174 | 3.80E-02 |
| regulation of cell-substrate adhesion (GO:0010810) | 8 | 8.695652174 | 3.80E-02 |
| axonogenesis (GO:0007409) | 10 | 10.86956522 | 4.21E-02 |
| glycerophospholipid metabolic process (GO:0006650) | 10 | 10.86956522 | 4.32E-02 |
| plasma membrane bounded cell projection organization (GO:0120036) | 17 | 18.47826087 | 4.58E-02 |
| regulation of biosynthetic process (GO:0009889) | 38 | 41.30434783 | 4.59E-02 |
| cellular response to oxygen-containing compound (GO:1901701) | 16 | 17.39130435 | 4.83E-02 |
| regulation of ERK1 and ERK2 cascade (GO:0070372) | 9 | 9.782608696 | 4.90E-02 |
