# Supplementary material for: Predisposing deleterious variants in the cancer-associated human kinases in the global populations
Source: PLoS One. 2024 Apr 18;19(4):e0298747. doi: 10.1371/journal.pone.0298747 (PMC11025791; doi:10.1371/journal.pone.0298747)
Supplement: S3 Table — (DOCX) [file pone.0298747.s005.docx]

**Supplementary Table S3:** Various molecular functions affected by the deleterious variants, as analyzed by the online Panther database.

| **Molecular function / GO ID** | **Count** | **%** | **Upload_1 (P-value)** |
| --- | --- | --- | --- |
| ribosomal protein S6 kinase activity (GO:0004711) | 3 | 3.260869565 | 2.61E-02 |
| transmembrane receptor protein tyrosine kinase activity (GO:0004714) | 16 | 17.39130435 | 2.03E-19 |
| histone kinase activity (GO:0035173) | 4 | 4.347826087 | 5.29E-03 |
| ephrin receptor activity (GO:0005003) | 4 | 4.347826087 | 7.78E-03 |
| phosphatidylinositol 3-kinase binding (GO:0043548) | 6 | 6.52173913 | 2.82E-05 |
| non-membrane spanning protein tyrosine kinase activity (GO:0004715) | 9 | 9.782608696 | 5.65E-09 |
| transmembrane receptor protein kinase activity (GO:0019199) | 16 | 17.39130435 | 8.37E-18 |
| protein tyrosine kinase activity (GO:0004713) | 33 | 35.86956522 | 2.01E-39 |
| tau-protein kinase activity (GO:0050321) | 4 | 4.347826087 | 1.30E-02 |
| protein serine/threonine/tyrosine kinase activity (GO:0004712) | 8 | 8.695652174 | 1.69E-07 |
| protein kinase activity (GO:0004672) | 83 | 90.2173913 | 7.73E-108 |
| protein serine/threonine kinase activity (GO:0004674) | 56 | 60.86956522 | 1.87E-63 |
| phosphotransferase activity, alcohol group as acceptor (GO:0016773) | 83 | 90.2173913 | 2.59E-101 |
| phosphatidylinositol-4,5-bisphosphate 3-kinase activity (GO:0046934) | 7 | 7.608695652 | 1.34E-04 |
| phosphatidylinositol 3-kinase activity (GO:0035004) | 8 | 8.695652174 | 1.52E-05 |
| kinase activity (GO:0016301) | 84 | 91.30434783 | 2.09E-100 |
| phosphatidylinositol bisphosphate kinase activity (GO:0052813) | 7 | 7.608695652 | 1.75E-04 |
| transferase activity, transferring phosphorus-containing groups (GO:0016772) | 84 | 91.30434783 | 1.57E-94 |
| magnesium ion binding (GO:0000287) | 16 | 17.39130435 | 1.04E-11 |
| phosphoprotein binding (GO:0051219) | 6 | 6.52173913 | 6.98E-03 |
| protein phosphatase binding (GO:0019903) | 8 | 8.695652174 | 6.45E-04 |
| ATP binding (GO:0005524) | 81 | 88.04347826 | 5.40E-76 |
| adenyl ribonucleotide binding (GO:0032559) | 81 | 88.04347826 | 1.05E-74 |
| adenyl nucleotide binding (GO:0030554) | 81 | 88.04347826 | 2.00E-74 |
| growth factor binding (GO:0019838) | 7 | 7.608695652 | 9.71E-03 |
| phosphatase binding (GO:0019902) | 9 | 9.782608696 | 4.22E-04 |
| drug binding (GO:0008144) | 81 | 88.04347826 | 1.27E-70 |
| purine ribonucleoside triphosphate binding (GO:0035639) | 81 | 88.04347826 | 7.19E-69 |
| purine ribonucleotide binding (GO:0032555) | 81 | 88.04347826 | 1.12E-67 |
| purine nucleotide binding (GO:0017076) | 81 | 88.04347826 | 2.06E-67 |
| ribonucleotide binding (GO:0032553) | 81 | 88.04347826 | 2.06E-67 |
| protein C-terminus binding (GO:0008022) | 8 | 8.695652174 | 1.05E-02 |
| Ras guanyl-nucleotide exchange factor activity (GO:0005088) | 10 | 10.86956522 | 7.07E-04 |
| nucleotide binding (GO:0000166) | 81 | 88.04347826 | 1.99E-63 |
| nucleoside phosphate binding (GO:1901265) | 81 | 88.04347826 | 2.06E-63 |
| carbohydrate derivative binding (GO:0097367) | 81 | 88.04347826 | 2.66E-62 |
| catalytic activity, acting on a protein (GO:0140096) | 86 | 93.47826087 | 2.72E-69 |
| transferase activity (GO:0016740) | 86 | 93.47826087 | 1.09E-68 |
| small molecule binding (GO:0036094) | 81 | 88.04347826 | 6.49E-58 |
| guanyl-nucleotide exchange factor activity (GO:0005085) | 10 | 10.86956522 | 7.05E-03 |
| anion binding (GO:0043168) | 81 | 88.04347826 | 1.06E-54 |
| Ras GTPase binding (GO:0017016) | 12 | 13.04347826 | 1.48E-02 |
| **Molecular function / GO ID** | **Count** | **%** | **Upload_1 (P-value)** |
| small GTPase binding (GO:0031267) | 12 | 13.04347826 | 1.94E-02 |
| protein domain specific binding (GO:0019904) | 13 | 14.13043478 | 3.68E-02 |
| catalytic activity (GO:0003824) | 87 | 94.56521739 | 3.08E-37 |
| heterocyclic compound binding (GO:1901363) | 84 | 91.30434783 | 9.75E-33 |
| organic cyclic compound binding (GO:0097159) | 84 | 91.30434783 | 3.02E-32 |
| ion binding (GO:0043167) | 84 | 91.30434783 | 1.70E-31 |
| molecular transducer activity (GO:0060089) | 21 | 22.82608696 | 1.04E-02 |
| identical protein binding (GO:0042802) | 23 | 25 | 5.96E-03 |
| enzyme binding (GO:0019899) | 29 | 31.52173913 | 2.18E-04 |
| protein binding (GO:0005515) | 83 | 90.2173913 | 5.04E-09 |
| binding (GO:0005488) | 90 | 97.82608696 | 2.55E-07 |
| Unclassified (UNCLASSIFIED) | 2 | 2.173913043 | 0.00E00 |
