# Supplementary material for: Predisposing deleterious variants in the cancer-associated human kinases in the global populations
Source: PLoS One. 2024 Apr 18;19(4):e0298747. doi: 10.1371/journal.pone.0298747 (PMC11025791; doi:10.1371/journal.pone.0298747)
Supplement: S4 Table — (DOCX) [file pone.0298747.s006.docx]

**Supplementary Table S4:** Cellular components affected by the deleterious variants (as analyzed by the online Panther database).

| **Cellular component / GO ID** | **Count** | **%** | **Upload_1 (P-value)** |
| --- | --- | --- | --- |
| basal plasma membrane (GO:0009925) | 4 | 4.347826087 | 3.15E-02 |
| protein kinase complex (GO:1902911) | 7 | 7.608695652 | 9.43E-04 |
| cytoplasmic side of plasma membrane (GO:0009898) | 7 | 7.608695652 | 9.08E-03 |
| receptor complex (GO:0043235) | 18 | 19.56521739 | 3.40E-10 |
| cytoplasmic side of membrane (GO:0098562) | 7 | 7.608695652 | 2.34E-02 |
| transferase complex, transferring phosphorus-containing groups (GO:0061695) | 10 | 10.86956522 | 4.15E-04 |
| nuclear body (GO:0016604) | 16 | 17.39130435 | 3.05E-04 |
| nucleoplasm part (GO:0044451) | 18 | 19.56521739 | 2.27E-03 |
| neuron projection (GO:0043005) | 18 | 19.56521739 | 2.14E-02 |
| plasma membrane part (GO:0044459) | 29 | 31.52173913 | 1.67E-02 |
| nucleoplasm (GO:0005654) | 34 | 36.95652174 | 4.59E-03 |
| cytosol (GO:0005829) | 45 | 48.91304348 | 4.25E-04 |
| nuclear lumen (GO:0031981) | 36 | 39.13043478 | 2.19E-02 |
| nucleus (GO:0005634) | 59 | 64.13043478 | 2.52E-05 |
| cytoplasm (GO:0005737) | 70 | 76.08695652 | 4.92E-02 |
| organelle (GO:0043226) | 81 | 88.04347826 | 7.04E-04 |
| intracellular organelle (GO:0043229) | 76 | 82.60869565 | 1.15E-02 |
| membrane-bounded organelle (GO:0043227) | 74 | 80.43478261 | 3.58E-02 |
| intracellular (GO:0005622) | 82 | 89.13043478 | 1.06E-02 |
| intracellular part (GO:0044424) | 82 | 89.13043478 | 1.06E-02 |
| cell part (GO:0044464) | 89 | 96.73913043 | 1.15E-02 |
| cell (GO:0005623) | 89 | 96.73913043 | 1.77E-02 |
| Unclassified (UNCLASSIFIED) | 2 | 2.173913043 | 0.00E00 |
