# Supplementary material for: Predisposing deleterious variants in the cancer-associated human kinases in the global populations
Source: PLoS One. 2024 Apr 18;19(4):e0298747. doi: 10.1371/journal.pone.0298747 (PMC11025791; doi:10.1371/journal.pone.0298747)
Supplement: S5 Table — (DOCX) [file pone.0298747.s007.docx]

**[Supplementary Table S](https://www.nature.com/articles/jhg201761#s1)5:** The total number of pathways associated with variants involved in carcinogenesis

| **Pathway Identifier** | **Pathway Name** | **Entities p-Value** | **Submitted entities found** |
| --- | --- | --- | --- |
| R-HSA-1839130 | Signaling by activated point mutants of FGFR3 | 9.08E-04 | FGFR3 |
| R-HSA-2033514 | FGFR3 mutant receptor activation | 0.001590949 | FGFR3 |
| R-HSA-187042 | TRKA activation by NGF | 0.011129904 | NTRK1 |
| R-HSA-190239 | FGFR3 ligand binding and activation | 0.017778563 | FGFR3 |
| R-HSA-187015 | Activation of TRKA receptors | 0.020523118 | NTRK1 |
| R-HSA-9036866 | Expression and Processing of Neurotrophins | 0.035821162 |  |
| R-HSA-167060 | NGF processing | 0.035821162 |  |
| R-HSA-5654704 | SHC-mediated cascade:FGFR3 | 0.040249136 | FGFR3 |
| R-HSA-169893 | Prolonged ERK activation events | 0.05928807 | NTRK1 |
| R-HSA-8853338 | Signaling by FGFR3 point mutants in cancer | 0.063048802 | FGFR3 |
| R-HSA-5655332 | Signaling by FGFR3 in disease | 0.063048802 | FGFR3 |
| R-HSA-5654227 | Phospholipase C-mediated cascade; FGFR3 | 0.078419572 | FGFR3 |
| R-HSA-6804115 | TP53 regulates transcription of additional cell cycle genes whose exact role in the p53 pathway remain uncertain | 0.080783883 | PLK2 |
| R-HSA-187687 | Signalling to ERKs | 0.083170284 | NTRK1 |
| R-HSA-3928665 | EPH-ephrin mediated repulsion of cells | 0.091689491 | YES1;EPHB4 |
| R-HSA-74751 | Insulin receptor signalling cascade | 0.100346382 | INSR;FGFR3 |
| R-HSA-77387 | Insulin receptor recycling | 0.110951696 | INSR |
| R-HSA-2033515 | t(4;14) translocations of FGFR3 | 0.114552591 | FGFR3 |
| R-HSA-8939242 | RUNX1 regulates transcription of genes involved in differentiation of keratinocytes | 0.118139091 |  |
| R-HSA-74752 | Signaling by Insulin receptor | 0.122548004 | INSR;FGFR3 |
| R-HSA-6811558 | PI5P, PP2A and IER3 Regulate PI3K/AKT Signaling | 0.123435842 | INSR;FGFR3 |
| R-HSA-74713 | IRS activation | 0.128812781 | INSR |
| R-HSA-187706 | Signalling to p38 via RIT and RIN | 0.128812781 | NTRK1 |
| R-HSA-9027283 | Erythropoietin activates STAT5 | 0.135857629 | JAK2 |
| R-HSA-8865999 | MET activates PTPN11 | 0.135857629 |  |
| R-HSA-1226099 | Signaling by FGFR in disease | 0.145429814 | FGFR3 |
| R-HSA-190371 | FGFR3b ligand binding and activation | 0.146319602 | FGFR3 |
| R-HSA-199418 | Negative regulation of the PI3K/AKT network | 0.148266553 | INSR;FGFR3 |
| R-HSA-6788467 | IL-6-type cytokine receptor ligand interactions | 0.166869915 | JAK2 |
| R-HSA-187024 | NGF-independant TRKA activation | 0.166869915 | NTRK1 |
| R-HSA-190372 | FGFR3c ligand binding and activation | 0.180298063 | FGFR3 |
| R-HSA-9022707 | MECP2 regulates transcription factors | 0.186931801 |  |
| R-HSA-8943723 | Regulation of PTEN mRNA translation | 0.186931801 |  |
| R-HSA-109704 | PI3K Cascade | 0.190208539 | FGFR3 |
| R-HSA-75953 | RNA Polymerase II Transcription Initiation | 0.190228771 | TAF1L |
| R-HSA-163680 | AMPK inhibits chREBP transcriptional activation activity | 0.190228771 |  |
| R-HSA-141430 | Inactivation of APC/C via direct inhibition of the APC/C complex | 0.200040713 |  |
| R-HSA-141405 | Inhibition of the proteolytic activity of APC/C required for the onset of anaphase by mitotic spindle checkpoint components | 0.200040713 |  |
| R-HSA-73779 | RNA Polymerase II Transcription Pre-Initiation And Promoter Opening | 0.206516718 | TAF1L |
| R-HSA-5654710 | PI-3K cascade:FGFR3 | 0.209326113 | FGFR3 |
| R-HSA-912526 | Interleukin receptor SHC signaling | 0.209735289 | JAK2 |
| R-HSA-5673001 | RAF/MAP kinase cascade | 0.212542087 | JAK2;FGFR3 |
| R-HSA-5654732 | Negative regulation of FGFR3 signaling | 0.21525066 | FGFR3 |
| R-HSA-199920 | CREB phosphorylation | 0.219313886 |  |
| R-HSA-5654706 | FRS-mediated FGFR3 signaling | 0.219704791 | FGFR3 |
| R-HSA-167161 | HIV Transcription Initiation | 0.222481215 | TAF1L |
| R-HSA-167162 | RNA Polymerase II HIV Promoter Escape | 0.222481215 | TAF1L |
| R-HSA-2219530 | Constitutive Signaling by Aberrant PI3K in Cancer | 0.22267884 | FGFR3 |
| R-HSA-112399 | IRS-mediated signalling | 0.231620483 | FGFR3 |
| R-HSA-170984 | ARMS-mediated activation | 0.231907301 | NTRK1 |
| R-HSA-167021 | PLC-gamma1 signalling | 0.241220577 | NTRK1 |
| R-HSA-390522 | Striated Muscle Contraction | 0.244300164 | TTN |
| R-HSA-1251932 | PLCG1 events in ERBB2 signaling | 0.244300164 |  |
| R-HSA-9034793 | Activated NTRK3 signals through PLCG1 | 0.244300164 |  |
| R-HSA-2428928 | IRS-related events triggered by IGF1R | 0.246573937 | FGFR3 |
| R-HSA-9026527 | Activated NTRK2 signals through PLCG1 | 0.247367415 |  |
| R-HSA-8934903 | Receptor Mediated Mitophagy | 0.247367415 |  |
| R-HSA-2428924 | IGF1R signaling cascade | 0.251068807 | FGFR3 |
| R-HSA-2404192 | Signaling by Type 1 Insulin-like Growth Factor 1 Receptor (IGF1R) | 0.252567747 | FGFR3 |
| R-HSA-74749 | Signal attenuation | 0.25649563 | INSR |
| R-HSA-2219528 | PI3K/AKT Signaling in Cancer | 0.258664861 | RPS6KB2;FGFR3 |
| R-HSA-212718 | EGFR interacts with phospholipase C-gamma | 0.259514018 |  |
| R-HSA-442720 | CREB1 phosphorylation through the activation of Adenylate Cyclase | 0.259514018 |  |
| R-HSA-198693 | AKT phosphorylates targets in the nucleus | 0.26252031 | RPS6KB2 |
| R-HSA-5673000 | RAF activation | 0.2684968 | JAK2 |
| R-HSA-177504 | Retrograde neurotrophin signalling | 0.2684968 | NTRK1 |
| R-HSA-167044 | Signalling to RAS | 0.271467094 | NTRK1 |
| R-HSA-3928662 | EPHB-mediated forward signaling | 0.275071033 | YES1;EPHB4 |
| R-HSA-111931 | PKA-mediated phosphorylation of CREB | 0.280306731 |  |
| R-HSA-380972 | Energy dependent regulation of mTOR by LKB1-AMPK | 0.280306731 |  |
| R-HSA-8948747 | Regulation of PTEN localization | 0.28904049 |  |
| R-HSA-193692 | Regulated proteolysis of p75NTR | 0.28904049 |  |
| R-HSA-73776 | RNA Polymerase II Promoter Escape | 0.291928431 | TAF1L |
| R-HSA-389513 | CTLA4 inhibitory signaling | 0.291928431 | YES1 |
| R-HSA-1170546 | Prolactin receptor signaling | 0.294804792 | JAK2 |
| R-HSA-3928663 | EPHA-mediated growth cone collapse | 0.297669621 | YES1 |
| R-HSA-5684996 | MAPK1/MAPK3 signaling | 0.298272142 | JAK2;FGFR3 |
| R-HSA-8964616 | G beta:gamma signalling through CDC42 | 0.300522961 |  |
| R-HSA-112411 | MAPK1 (ERK2) activation | 0.309014511 | JAK2 |
| R-HSA-877312 | Regulation of IFNG signaling | 0.309014511 | JAK2 |
| R-HSA-110056 | MAPK3 (ERK1) activation | 0.311822353 | JAK2 |
| R-HSA-6802946 | Signaling by moderate kinase activity BRAF mutants | 0.311822353 | JAK2 |
| R-HSA-76042 | RNA Polymerase II Transcription Initiation And Promoter Clearance | 0.311822353 | TAF1L |
| R-HSA-6802955 | Paradoxical activation of RAF signaling by kinase inactive BRAF | 0.314618934 | JAK2 |
| R-HSA-8849472 | PTK6 Down-Regulation | 0.317404296 |  |
| R-HSA-198725 | Nuclear Events (kinase and transcription factor activation) | 0.317404296 |  |
| R-HSA-170968 | Frs2-mediated activation | 0.320178486 | NTRK1 |
| R-HSA-1234158 | Regulation of gene expression by Hypoxia-inducible Factor | 0.325693522 |  |
| R-HSA-6802952 | Signaling by BRAF and RAF fusions | 0.328434457 | JAK2 |
| R-HSA-180336 | SHC1 events in EGFR signaling | 0.331164395 |  |
| R-HSA-9027284 | Erythropoietin activates RAS | 0.333883378 | JAK2 |
| R-HSA-937042 | IRAK2 mediated activation of TAK1 complex | 0.333883378 |  |
| R-HSA-912631 | Regulation of signaling by CBL | 0.336591452 | YES1 |
| R-HSA-442729 | CREB1 phosphorylation through the activation of CaMKII/CaMKK/CaMKIV cascasde | 0.336591452 |  |
| R-HSA-1250347 | SHC1 events in ERBB4 signaling | 0.339288658 |  |
| R-HSA-9014325 | TICAM1,TRAF6-dependent induction of TAK1 complex | 0.339288658 |  |
| R-HSA-111932 | CaMK IV-mediated phosphorylation of CREB | 0.34197504 |  |
| R-HSA-205043 | NRIF signals cell death from the nucleus | 0.344650641 |  |
| R-HSA-1433557 | Signaling by SCF-KIT | 0.345113753 | YES1;JAK2 |
| R-HSA-6802949 | Signaling by RAS mutants | 0.347315503 | JAK2 |
| R-HSA-442742 | CREB1 phosphorylation through NMDA receptor-mediated activation of RAS signaling | 0.347315503 |  |
| R-HSA-975163 | IRAK2 mediated activation of TAK1 complex upon TLR7/8 or 9 stimulation | 0.349969669 |  |
| R-HSA-5625900 | RHO GTPases activate CIT | 0.349969669 |  |
| R-HSA-912694 | Regulation of IFNA signaling | 0.352613182 |  |
| R-HSA-937072 | TRAF6-mediated induction of TAK1 complex within TLR4 complex | 0.352613182 |  |
| R-HSA-9027277 | Erythropoietin activates Phospholipase C gamma (PLCG) | 0.355246083 | JAK2 |
| R-HSA-198745 | Signalling to STAT3 | 0.357868414 | NTRK1 |
| R-HSA-512988 | Interleukin-3, Interleukin-5 and GM-CSF signaling | 0.361251359 | YES1;JAK2 |
| R-HSA-5205685 | Pink/Parkin Mediated Mitophagy | 0.363081537 |  |
| R-HSA-456926 | Thrombin signalling through proteinase activated receptors (PARs) | 0.365672411 |  |
| R-HSA-5654228 | Phospholipase C-mediated cascade; FGFR4 | 0.37338278 |  |
| R-HSA-9020558 | Interleukin-2 signaling | 0.37338278 |  |
| R-HSA-8854214 | TBC/RABGAPs | 0.378471559 |  |
| R-HSA-5654219 | Phospholipase C-mediated cascade: FGFR1 | 0.381000631 |  |
| R-HSA-1250196 | SHC1 events in ERBB2 signaling | 0.381000631 |  |
| R-HSA-5654221 | Phospholipase C-mediated cascade; FGFR2 | 0.388527059 |  |
| R-HSA-418886 | Netrin mediated repulsion signals | 0.393494423 |  |
| R-HSA-2514859 | Inactivation, recovery and regulation of the phototransduction cascade | 0.40087088 | GUCY2D |
| R-HSA-210990 | PECAM1 interactions | 0.40087088 | YES1 |
| R-HSA-372708 | p130Cas linkage to MAPK signaling for integrins | 0.40087088 |  |
| R-HSA-936964 | Activation of IRF3/IRF7 mediated by TBK1/IKK epsilon | 0.40087088 |  |
| R-HSA-9022699 | MECP2 regulates neuronal receptors and channels | 0.40087088 |  |
| R-HSA-432142 | Platelet sensitization by LDL | 0.403309966 |  |
| R-HSA-1433559 | Regulation of KIT signaling | 0.405739251 | YES1 |
| R-HSA-9615017 | FOXO-mediated transcription of oxidative stress, metabolic and neuronal genes | 0.410568573 |  |
| R-HSA-6802957 | Oncogenic MAPK signaling | 0.412968687 | JAK2 |
| R-HSA-2514856 | The phototransduction cascade | 0.417740014 | GUCY2D |
| R-HSA-69231 | Cyclin D associated events in G1 | 0.417740014 | JAK2 |
| R-HSA-69236 | G1 Phase | 0.417740014 | JAK2 |
| R-HSA-416482 | G alpha (12/13) signalling events | 0.417740014 | TRIO |
| R-HSA-5336415 | Uptake and function of diphtheria toxin | 0.422473059 |  |
| R-HSA-430116 | GP1b-IX-V activation signalling | 0.424825321 |  |
| R-HSA-1059683 | Interleukin-6 signaling | 0.427168126 | JAK2 |
| R-HSA-166208 | mTORC1-mediated signalling | 0.431825516 |  |
| R-HSA-9027276 | Erythropoietin activates Phosphoinositide-3-kinase (PI3K) | 0.438741608 | JAK2 |
| R-HSA-112409 | RAF-independent MAPK1/3 activation | 0.441028455 | JAK2 |
| R-HSA-982772 | Growth hormone receptor signaling | 0.441028455 | JAK2 |
| R-HSA-8849474 | PTK6 Activates STAT3 | 0.441028455 |  |
| R-HSA-8849471 | PTK6 Regulates RHO GTPases, RAS GTPase and MAP kinases | 0.441028455 |  |
| R-HSA-8866652 | Synthesis of active ubiquitin: roles of E1 and E2 enzymes | 0.445574594 |  |
| R-HSA-418890 | Role of second messengers in netrin-1 signaling | 0.454557668 |  |
| R-HSA-2029481 | FCGR activation | 0.456780896 | YES1 |
| R-HSA-8849468 | PTK6 Regulates Proteins Involved in RNA Processing | 0.456780896 |  |
| R-HSA-111933 | Calmodulin induced events | 0.456780896 |  |
| R-HSA-6791312 | TP53 Regulates Transcription of Cell Cycle Genes | 0.459921337 | PLK2 |
| R-HSA-111997 | CaM pathway | 0.461200554 |  |
| R-HSA-209543 | p75NTR recruits signalling complexes | 0.465584716 |  |
| R-HSA-445355 | Smooth Muscle Contraction | 0.465584716 |  |
| R-HSA-8853659 | RET signaling | 0.467763575 |  |
| R-HSA-5654708 | Downstream signaling of activated FGFR3 | 0.469323502 | FGFR3 |
| R-HSA-2586552 | Signaling by Leptin | 0.472095021 | JAK2 |
| R-HSA-198203 | PI3K/AKT activation | 0.472095021 | NTRK1 |
| R-HSA-5638303 | Inhibition of Signaling by Overexpressed EGFR | 0.476391671 |  |
| R-HSA-5638302 | Signaling by Overexpressed Wild-Type EGFR in Cancer | 0.476391671 |  |
| R-HSA-8985947 | Interleukin-9 signaling | 0.476391671 |  |
| R-HSA-8853334 | Signaling by FGFR3 fusions in cancer | 0.480653801 | FGFR3 |
| R-HSA-5099900 | WNT5A-dependent internalization of FZD4 | 0.480653801 |  |
| R-HSA-9020933 | Interleukin-23 signaling | 0.482772006 | JAK2 |
| R-HSA-141444 | Amplification of signal from unattached kinetochores via a MAD2 inhibitory signal | 0.482772006 |  |
| R-HSA-141424 | Amplification of signal from the kinetochores | 0.482772006 |  |
| R-HSA-167172 | Transcription of the HIV genome | 0.491159891 | TAF1L |
| R-HSA-5205647 | Mitophagy | 0.493235796 |  |
| R-HSA-9006934 | Signaling by Receptor Tyrosine Kinases | 0.494157831 | NTRK1;YES1;AXL;INSR;JAK2;FGFR3 |
| R-HSA-6783589 | Interleukin-6 family signaling | 0.495303342 | JAK2 |
| R-HSA-8876198 | RAB GEFs exchange GTP for GDP on RABs | 0.495303342 |  |
| R-HSA-194840 | Rho GTPase cycle | 0.499413487 | TRIO |
| R-HSA-210993 | Tie2 Signaling | 0.503490592 |  |
| R-HSA-9614657 | FOXO-mediated transcription of cell death genes | 0.507534918 |  |
| R-HSA-114608 | Platelet degranulation | 0.50954487 | TTN |
| R-HSA-5218921 | VEGFR2 mediated cell proliferation | 0.515526271 |  |
| R-HSA-8934593 | Regulation of RUNX1 Expression and Activity | 0.515526271 |  |
| R-HSA-5635838 | Activation of SMO | 0.519473813 |  |
| R-HSA-193639 | p75NTR signals via NF-kB | 0.519473813 |  |
| R-HSA-5620912 | Anchoring of the basal body to the plasma membrane | 0.521435662 | MARK4 |
| R-HSA-418889 | Caspase activation via Dependence Receptors in the absence of ligand | 0.523389604 | DAPK3 |
| R-HSA-9603381 | Activated NTRK3 signals through PI3K | 0.525335672 |  |
| R-HSA-2179392 | EGFR Transactivation by Gastrin | 0.533041822 |  |
| R-HSA-1251985 | Nuclear signaling by ERBB4 | 0.533041822 |  |
| R-HSA-1266695 | Interleukin-7 signaling | 0.534948985 |  |
| R-HSA-69618 | Mitotic Spindle Checkpoint | 0.536848458 |  |
| R-HSA-450282 | MAPK targets/ Nuclear events mediated by MAP kinases | 0.538740274 |  |
| R-HSA-180292 | GAB1 signalosome | 0.542501054 |  |
| R-HSA-9617828 | FOXO-mediated transcription of cell cycle genes | 0.544370078 |  |
| R-HSA-6804760 | Regulation of TP53 Activity through Methylation | 0.54993205 |  |
| R-HSA-2262749 | Cellular response to hypoxia | 0.54993205 |  |
| R-HSA-1234174 | Regulation of Hypoxia-inducible Factor (HIF) by oxygen | 0.54993205 |  |
| R-HSA-111996 | Ca-dependent events | 0.551771107 |  |
| R-HSA-5655291 | Signaling by FGFR4 in disease | 0.555426997 |  |
| R-HSA-844456 | The NLRP3 inflammasome | 0.555426997 |  |
| R-HSA-1295596 | Spry regulation of FGF signaling | 0.55724389 |  |
| R-HSA-176408 | Regulation of APC/C activators between G1/S and early anaphase | 0.559053453 |  |
| R-HSA-8984722 | Interleukin-35 Signalling | 0.562650708 | JAK2 |
| R-HSA-187037 | Signaling by NTRK1 (TRKA) | 0.567248685 | NTRK1 |
| R-HSA-9028731 | Activated NTRK2 signals through FRS2 and FRS3 | 0.567992348 |  |
| R-HSA-622312 | Inflammasomes | 0.567992348 |  |
| R-HSA-5628897 | TP53 Regulates Metabolic Genes | 0.569758544 |  |
| R-HSA-9006335 | Signaling by Erythropoietin | 0.571517613 | JAK2 |
| R-HSA-5654720 | PI-3K cascade:FGFR4 | 0.576752339 |  |
| R-HSA-1839117 | Signaling by cytosolic FGFR1 fusion mutants | 0.578483181 |  |
| R-HSA-8854691 | Interleukin-20 family signaling | 0.580207036 | JAK2 |
| R-HSA-5357769 | Caspase activation via extrinsic apoptotic signalling pathway | 0.581923933 | DAPK3 |
| R-HSA-9020956 | Interleukin-27 signaling | 0.581923933 | JAK2 |
| R-HSA-5654689 | PI-3K cascade:FGFR1 | 0.581923933 |  |
| R-HSA-5654733 | Negative regulation of FGFR4 signaling | 0.583633898 |  |
| R-HSA-4570464 | SUMOylation of RNA binding proteins | 0.587033147 |  |
| R-HSA-5654695 | PI-3K cascade:FGFR2 | 0.587033147 |  |
| R-HSA-5654712 | FRS-mediated FGFR4 signaling | 0.588722484 |  |
| R-HSA-6804756 | Regulation of TP53 Activity through Phosphorylation | 0.593749679 | TAF1L |
| R-HSA-5654693 | FRS-mediated FGFR1 signaling | 0.593749679 |  |
| R-HSA-5654727 | Negative regulation of FGFR2 signaling | 0.593749679 |  |
| R-HSA-5654726 | Negative regulation of FGFR1 signaling | 0.593749679 |  |
| R-HSA-418885 | DCC mediated attractive signaling | 0.595411894 | TRIO |
| R-HSA-5654700 | FRS-mediated FGFR2 signaling | 0.598716212 |  |
| R-HSA-2565942 | Regulation of PLK1 Activity at G2/M Transition | 0.600358368 |  |
| R-HSA-5663202 | Diseases of signal transduction | 0.603584119 | RPS6KB2;JAK2;FGFR3 |
| R-HSA-174143 | APC/C-mediated degradation of cell cycle proteins | 0.610072929 |  |
| R-HSA-453276 | Regulation of mitotic cell cycle | 0.610072929 |  |
| R-HSA-5654741 | Signaling by FGFR3 | 0.612290074 | FGFR3 |
| R-HSA-375165 | NCAM signaling for neurite out-growth | 0.61484243 |  |
| R-HSA-1839124 | FGFR1 mutant receptor activation | 0.616419431 |  |
| R-HSA-674695 | RNA Polymerase II Pre-transcription Events | 0.61799006 | TAF1L |
| R-HSA-5683057 | MAPK family signaling cascades | 0.620484396 | JAK2;FGFR3 |
| R-HSA-1257604 | PIP3 activates AKT signaling | 0.627215653 | INSR;RPS6KB2;FGFR3 |
| R-HSA-1912408 | Pre-NOTCH Transcription and Translation | 0.631843071 |  |
| R-HSA-450294 | MAP kinase activation | 0.633351347 |  |
| R-HSA-5655253 | Signaling by FGFR2 in disease | 0.634853523 |  |
| R-HSA-445144 | Signal transduction by L1 | 0.637839676 |  |
| R-HSA-9007101 | Rab regulation of trafficking | 0.637839676 |  |
| R-HSA-76005 | Response to elevated platelet cytosolic Ca2+ | 0.639323701 | TTN |
| R-HSA-8940973 | RUNX2 regulates osteoblast differentiation | 0.640801723 | YES1 |
| R-HSA-1660499 | Synthesis of PIPs at the plasma membrane | 0.645200014 |  |
| R-HSA-448424 | Interleukin-17 signaling | 0.645200014 |  |
| R-HSA-193648 | NRAGE signals death through JNK | 0.653837735 | TRIO |
| R-HSA-881907 | Gastrin-CREB signalling pathway via PKC and MAPK | 0.653837735 |  |
| R-HSA-114604 | GPVI-mediated activation cascade | 0.655257035 |  |
| R-HSA-69656 | Cyclin A:Cdk2-associated events at S phase entry | 0.659480563 |  |
| R-HSA-1489509 | DAG and IP3 signaling | 0.662267834 |  |
| R-HSA-418346 | Platelet homeostasis | 0.662267834 |  |
| R-HSA-397795 | G-protein beta:gamma signalling | 0.663653012 |  |
| R-HSA-2871837 | FCERI mediated NF-kB activation | 0.669137872 |  |
| R-HSA-1236382 | Constitutive Signaling by Ligand-Responsive EGFR Cancer Variants | 0.673193494 |  |
| R-HSA-5637815 | Signaling by Ligand-Responsive EGFR Variants in Cancer | 0.673193494 |  |
| R-HSA-2151201 | Transcriptional activation of mitochondrial biogenesis | 0.677200041 |  |
| R-HSA-2187338 | Visual phototransduction | 0.688931057 | GUCY2D |
| R-HSA-202433 | Generation of second messenger molecules | 0.688931057 |  |
| R-HSA-438064 | Post NMDA receptor activation events | 0.688931057 |  |
| R-HSA-389356 | CD28 co-stimulation | 0.69020824 | YES1 |
| R-HSA-9034015 | Signaling by NTRK3 (TRKC) | 0.69020824 |  |
| R-HSA-112043 | PLC beta mediated events | 0.691480246 |  |
| R-HSA-5655302 | Signaling by FGFR1 in disease | 0.692747097 |  |
| R-HSA-112040 | G-protein mediated events | 0.692747097 |  |
| R-HSA-8941326 | RUNX2 regulates bone development | 0.694008813 | YES1 |
| R-HSA-8852135 | Protein ubiquitination | 0.694008813 |  |
| R-HSA-2029485 | Role of phospholipids in phagocytosis | 0.70024109 |  |
| R-HSA-1912422 | Pre-NOTCH Expression and Processing | 0.70024109 |  |
| R-HSA-442755 | Activation of NMDA receptors and postsynaptic events | 0.70024109 |  |
| R-HSA-3214858 | RMTs methylate histone arginines | 0.709953102 | JAK2 |
| R-HSA-190236 | Signaling by FGFR | 0.710160959 | FGFR3 |
| R-HSA-69202 | Cyclin E associated events during G1/S transition | 0.713514469 |  |
| R-HSA-9006925 | Intracellular signaling by second messengers | 0.715468303 | INSR;RPS6KB2;FGFR3 |
| R-HSA-5674400 | Constitutive Signaling by AKT1 E17K in Cancer | 0.721657342 | RPS6KB2 |
| R-HSA-4086400 | PCP/CE pathway | 0.721657342 |  |
| R-HSA-8983432 | Interleukin-15 signaling | 0.722801804 |  |
| R-HSA-5339562 | Uptake and actions of bacterial toxins | 0.725076814 |  |
| R-HSA-186763 | Downstream signal transduction | 0.728454817 |  |
| R-HSA-354192 | Integrin alphaIIb beta3 signaling | 0.745790687 |  |
| R-HSA-9006921 | Integrin signaling | 0.745790687 |  |
| R-HSA-204998 | Cell death signalling via NRAGE, NRIF and NADE | 0.752006024 | TRIO |
| R-HSA-1483255 | PI Metabolism | 0.752006024 |  |
| R-HSA-5689896 | Ovarian tumor domain proteases | 0.753027228 |  |
| R-HSA-163685 | Integration of energy metabolism | 0.759067871 |  |
| R-HSA-9020702 | Interleukin-1 signaling | 0.761048829 |  |
| R-HSA-202424 | Downstream TCR signaling | 0.762033271 |  |
| R-HSA-9006115 | Signaling by NTRK2 (TRKB) | 0.762033271 |  |
| R-HSA-76009 | Platelet Aggregation (Plug Formation) | 0.767856412 |  |
| R-HSA-186797 | Signaling by PDGF | 0.769766027 |  |
| R-HSA-6785807 | Interleukin-4 and Interleukin-13 signaling | 0.771660136 | JAK2 |
| R-HSA-165159 | mTOR signalling | 0.771660136 |  |
| R-HSA-8950505 | Gene and protein expression by JAK-STAT signaling after Interleukin-12 stimulation | 0.771660136 |  |
| R-HSA-5632684 | Hedgehog 'on' state | 0.774472498 |  |
| R-HSA-397014 | Muscle contraction | 0.779083982 | TTN |
| R-HSA-5637810 | Constitutive Signaling by EGFRvIII | 0.779995048 |  |
| R-HSA-5637812 | Signaling by EGFRvIII in Cancer | 0.779995048 |  |
| R-HSA-168643 | Nucleotide-binding domain, leucine rich repeat containing receptor (NLR) signaling pathways | 0.782706053 |  |
| R-HSA-166520 | Signaling by NTRKs | 0.782804677 | NTRK1 |
| R-HSA-388841 | Costimulation by the CD28 family | 0.784495044 | YES1 |
| R-HSA-416993 | Trafficking of GluR2-containing AMPA receptors | 0.792367257 |  |
| R-HSA-909733 | Interferon alpha/beta signaling | 0.794077713 |  |
| R-HSA-1236394 | Signaling by ERBB4 | 0.79577426 |  |
| R-HSA-1643713 | Signaling by EGFR in Cancer | 0.796617352 |  |
| R-HSA-5654716 | Downstream signaling of activated FGFR4 | 0.797457009 |  |
| R-HSA-1227986 | Signaling by ERBB2 | 0.801604244 | YES1 |
| R-HSA-5654696 | Downstream signaling of activated FGFR2 | 0.802423576 |  |
| R-HSA-5654687 | Downstream signaling of activated FGFR1 | 0.802423576 |  |
| R-HSA-451927 | Interleukin-2 family signaling | 0.803239568 | JAK2 |
| R-HSA-2682334 | EPH-Ephrin signaling | 0.806541911 | YES1;EPHB4 |
| R-HSA-5607764 | CLEC7A (Dectin-1) signaling | 0.810435404 |  |
| R-HSA-8866910 | TFAP2 (AP-2) family regulates transcription of growth factors and their receptors | 0.818126398 |  |
| R-HSA-975138 | TRAF6 mediated induction of NFkB and MAP kinases upon TLR7/8 or 9 activation | 0.818878345 |  |
| R-HSA-877300 | Interferon gamma signaling | 0.825509244 | JAK2 |
| R-HSA-975871 | MyD88 cascade initiated on plasma membrane | 0.826231054 |  |
| R-HSA-1592230 | Mitochondrial biogenesis | 0.826949916 |  |
| R-HSA-168142 | Toll Like Receptor 10 (TLR10) Cascade | 0.826949916 |  |
| R-HSA-168176 | Toll Like Receptor 5 (TLR5) Cascade | 0.826949916 |  |
| R-HSA-399719 | Trafficking of AMPA receptors | 0.831201861 |  |
| R-HSA-162599 | Late Phase of HIV Life Cycle | 0.832596131 | TAF1L |
| R-HSA-399721 | Glutamate binding, activation of AMPA receptors and synaptic plasticity | 0.833979032 |  |
| R-HSA-8948751 | Regulation of PTEN stability and activity | 0.835350657 |  |
| R-HSA-975155 | MyD88 dependent cascade initiated on endosome | 0.838060438 |  |
| R-HSA-168181 | Toll Like Receptor 7/8 (TLR7/8) Cascade | 0.838060438 |  |
| R-HSA-69242 | S Phase | 0.838730976 |  |
| R-HSA-168138 | Toll Like Receptor 9 (TLR9) Cascade | 0.842697039 |  |
| R-HSA-8986944 | Transcriptional Regulation by MECP2 | 0.843997544 |  |
| R-HSA-9020591 | Interleukin-12 signaling | 0.845928429 | JAK2 |
| R-HSA-69206 | G1/S Transition | 0.847202561 |  |
| R-HSA-6806834 | Signaling by MET | 0.847835722 |  |
| R-HSA-168164 | Toll Like Receptor 3 (TLR3) Cascade | 0.848466292 |  |
| R-HSA-162587 | HIV Life Cycle | 0.852195914 | TAF1L |
| R-HSA-2029480 | Fcgamma receptor (FCGR) dependent phagocytosis | 0.859385434 | YES1 |
| R-HSA-373752 | Netrin-1 signaling | 0.86284963 | TRIO |
| R-HSA-381038 | XBP1(S) activates chaperone genes | 0.867888565 |  |
| R-HSA-937061 | TRIF(TICAM1)-mediated TLR4 signaling | 0.868983268 |  |
| R-HSA-166166 | MyD88-independent TLR4 cascade | 0.868983268 |  |
| R-HSA-193704 | p75 NTR receptor-mediated signaling | 0.869527257 | TRIO |
| R-HSA-381070 | IRE1alpha activates chaperones | 0.870608555 |  |
| R-HSA-111885 | Opioid Signalling | 0.872213939 |  |
| R-HSA-5654743 | Signaling by FGFR4 | 0.87379966 |  |
| R-HSA-3858494 | Beta-catenin independent WNT signaling | 0.87379966 |  |
| R-HSA-202403 | TCR signaling | 0.87742454 |  |
| R-HSA-6791226 | Major pathway of rRNA processing in the nucleolus and cytosol | 0.879449556 | RIOK1 |
| R-HSA-5654736 | Signaling by FGFR1 | 0.879950628 |  |
| R-HSA-69620 | Cell Cycle Checkpoints | 0.882425321 |  |
| R-HSA-3928664 | Ephrin signaling | 0.882914183 | EPHB4 |
| R-HSA-416476 | G alpha (q) signalling events | 0.887693785 | TRIO |
| R-HSA-166058 | MyD88:MAL(TIRAP) cascade initiated on plasma membrane | 0.887693785 |  |
| R-HSA-168179 | Toll Like Receptor TLR1:TLR2 Cascade | 0.88955124 |  |
| R-HSA-168188 | Toll Like Receptor TLR6:TLR2 Cascade | 0.88955124 |  |
| R-HSA-5358351 | Signaling by Hedgehog | 0.890010845 |  |
| R-HSA-983231 | Factors involved in megakaryocyte development and platelet production | 0.890468563 | JAK2 |
| R-HSA-181438 | Toll Like Receptor 2 (TLR2) Cascade | 0.890924401 |  |
| R-HSA-177929 | Signaling by EGFR | 0.891830466 |  |
| R-HSA-5617833 | Cilium Assembly | 0.896682624 | MARK4 |
| R-HSA-8868773 | rRNA processing in the nucleus and cytosol | 0.902140605 | RIOK1 |
| R-HSA-446652 | Interleukin-1 family signaling | 0.905751485 |  |
| R-HSA-4420097 | VEGFA-VEGFR2 Pathway | 0.910736168 | AXL |
| R-HSA-5357905 | Regulation of TNFR1 signaling | 0.910736168 |  |
| R-HSA-5654738 | Signaling by FGFR2 | 0.910736168 |  |
| R-HSA-69275 | G2/M Transition | 0.912948138 |  |
| R-HSA-9614085 | FOXO-mediated transcription | 0.912948138 |  |
| R-HSA-453274 | Mitotic G2-G2/M phases | 0.91367337 |  |
| R-HSA-453279 | Mitotic G1-G1/S phases | 0.914033746 | JAK2 |
| R-HSA-8878166 | Transcriptional regulation by RUNX2 | 0.91616503 | YES1 |
| R-HSA-194138 | Signaling by VEGF | 0.917556825 | AXL |
| R-HSA-3247509 | Chromatin modifying enzymes | 0.918244145 | JAK2 |
| R-HSA-4839726 | Chromatin organization | 0.918244145 | JAK2 |
| R-HSA-9006927 | Signaling by Non-Receptor Tyrosine Kinases | 0.923221888 |  |
| R-HSA-8848021 | Signaling by PTK6 | 0.923221888 |  |
| R-HSA-447115 | Interleukin-12 family signaling | 0.925753118 | JAK2 |
| R-HSA-2454202 | Fc epsilon receptor (FCERI) signaling | 0.926063672 |  |
| R-HSA-5621481 | C-type lectin receptors (CLRs) | 0.926987664 |  |
| R-HSA-72312 | rRNA processing | 0.92939629 | RIOK1 |
| R-HSA-6807070 | PTEN Regulation | 0.935622993 |  |
| R-HSA-166016 | Toll Like Receptor 4 (TLR4) Cascade | 0.935892762 |  |
| R-HSA-75893 | TNF signaling | 0.941059784 |  |
| R-HSA-1483257 | Phospholipid metabolism | 0.944195608 |  |
| R-HSA-72163 | mRNA Splicing - Major Pathway | 0.951639523 |  |
| R-HSA-72172 | mRNA Splicing | 0.95421891 |  |
| R-HSA-168898 | Toll-like Receptor Cascades | 0.95536313 |  |
| R-HSA-112314 | Neurotransmitter receptors and postsynaptic signal transmission | 0.957924356 |  |
| R-HSA-373760 | L1CAM interactions | 0.961169683 |  |
| R-HSA-5688426 | Deubiquitination | 0.963863198 |  |
| R-HSA-8864260 | Transcriptional regulation by the AP-2 (TFAP2) family of transcription factors | 0.964469664 |  |
| R-HSA-381119 | Unfolded Protein Response (UPR) | 0.972095125 |  |
| R-HSA-76002 | Platelet activation, signaling and aggregation | 0.973027065 | TTN |
| R-HSA-157118 | Signaling by NOTCH | 0.973255209 |  |
| R-HSA-202733 | Cell surface interactions at the vascular wall | 0.973481447 | YES1 |
| R-HSA-109581 | Apoptosis | 0.974584613 | DAPK3 |
| R-HSA-5357801 | Programmed Cell Death | 0.976256179 | DAPK3 |
| R-HSA-72203 | Processing of Capped Intron-Containing Pre-mRNA | 0.976755935 |  |
| R-HSA-162906 | HIV Infection | 0.977819142 | TAF1L |
| R-HSA-195258 | RHO GTPase Effectors | 0.979013906 |  |
| R-HSA-3700989 | Transcriptional Regulation by TP53 | 0.979192457 | PLK2;TAF1L |
| R-HSA-913531 | Interferon Signaling | 0.97980361 | JAK2 |
| R-HSA-1852241 | Organelle biogenesis and maintenance | 0.980646689 | MARK4 |
| R-HSA-8878171 | Transcriptional regulation by RUNX1 | 0.983332913 |  |
| R-HSA-422475 | Axon guidance | 0.984539424 | YES1;TRIO;EPHB4 |
| R-HSA-418594 | G alpha (i) signalling events | 0.987859519 | GUCY2D |
| R-HSA-194315 | Signaling by Rho GTPases | 0.988714015 | TRIO |
| R-HSA-5633007 | Regulation of TP53 Activity | 0.990164556 | TAF1L |
| R-HSA-73887 | Death Receptor Signalling | 0.990858643 | TRIO |
| R-HSA-112315 | Transmission across Chemical Synapses | 0.991897933 |  |
| R-HSA-109582 | Hemostasis | 0.994579231 | YES1;JAK2;TTN |
| R-HSA-2262752 | Cellular responses to stress | 0.995806125 |  |
| R-HSA-69278 | Cell Cycle, Mitotic | 0.996335716 | JAK2 |
| R-HSA-112316 | Neuronal System | 0.996785186 |  |
| R-HSA-8953897 | Cellular responses to external stimuli | 0.997664086 |  |
| R-HSA-195721 | Signaling by WNT | 0.998319819 |  |
| R-HSA-5663205 | Infectious disease | 0.998377981 | TAF1L |
| R-HSA-3108232 | SUMO E3 ligases SUMOylate target proteins | 0.99858518 |  |
| R-HSA-388396 | GPCR downstream signalling | 0.998670958 | GUCY2D;TRIO |
| R-HSA-449147 | Signaling by Interleukins | 0.998902737 | YES1;JAK2 |
| R-HSA-2990846 | SUMOylation | 0.998914773 |  |
| R-HSA-199991 | Membrane Trafficking | 0.999087065 |  |
| R-HSA-1643685 | Disease | 0.999128954 | RPS6KB2;TAF1L;JAK2;FGFR3 |
| R-HSA-5653656 | Vesicle-mediated transport | 0.999587325 |  |
| R-HSA-1640170 | Cell Cycle | 0.999659772 | JAK2 |
| R-HSA-372790 | Signaling by GPCR | 0.999776898 | GUCY2D;TRIO |
| R-HSA-1280218 | Adaptive Immune System | 0.999807298 | YES1 |
| R-HSA-8953854 | Metabolism of RNA | 0.99986475 | RIOK1 |
| R-HSA-1266738 | Developmental Biology | 0.999941843 | YES1;TRIO;EPHB4 |
| R-HSA-1280215 | Cytokine Signaling in Immune system | 0.999955555 | YES1;JAK2 |
| R-HSA-212436 | Generic Transcription Pathway | 0.999994187 | YES1;PLK2;TAF1L |
| R-HSA-168249 | Innate Immune System | 0.999994841 | YES1 |
| R-HSA-556833 | Metabolism of lipids | 0.999995795 |  |
| R-HSA-73857 | RNA Polymerase II Transcription | 0.999997663 | YES1;PLK2;TAF1L |
| R-HSA-162582 | Signal Transduction | 0.999998459 | NTRK1;GUCY2D;YES1;TRIO;AXL;INSR;RPS6KB2;JAK2;FGFR3 |
| R-HSA-74160 | Gene expression (Transcription) | 0.999999288 | YES1;PLK2;TAF1L |
| R-HSA-597592 | Post-translational protein modification | 0.999999934 |  |
| R-HSA-168256 | Immune System | 1 | YES1;JAK2 |
| R-HSA-392499 | Metabolism of proteins | 1 |  |
| R-HSA-1430728 | Metabolism | 1 |  |
